# Rapid Access to Potent Bispecific T Cell Engagers Using Biogenic Tyrosine Click Chemistry

**DOI:** 10.1021/acs.bioconjchem.3c00357

**Published:** 2023-11-14

**Authors:** Irene Shajan, Léa N. C. Rochet, Shannon R. Tracey, Bianka Jackowska, Rania Benazza, Oscar Hernandez-Alba, Sarah Cianférani, Christopher J. Scott, Floris L. van Delft, Vijay Chudasama, Bauke Albada

**Affiliations:** † Laboratory of Organic Chemistry, Wageningen University & Research, Stippeneng 4, Wageningen 6807 WE, The Netherlands; ‡ Department of Chemistry, University College London, 20 Gordon St, London WC1H 0AJ, U.K.; § Patrick G Johnston Centre for Cancer Research, School of Medicine, Dentistry and Biomedical Sciences, Queen’s University Belfast, 97 Lisburn Road, Belfast BT9 7BL, U.K.; ∥ Laboratoire de Spectrométrie de Masse BioOrganique, Université de Strasbourg, CNRS, IPHC UMR 7178, Strasbourg 67000, France; ⊥ Infrastructure Nationale de Protéomique ProFI − FR2048, Strasbourg 67087, France; # Synaffix BV − A Lonza Company, Kloosterstraat 9, Oss 5349 AB, The Netherlands

## Abstract

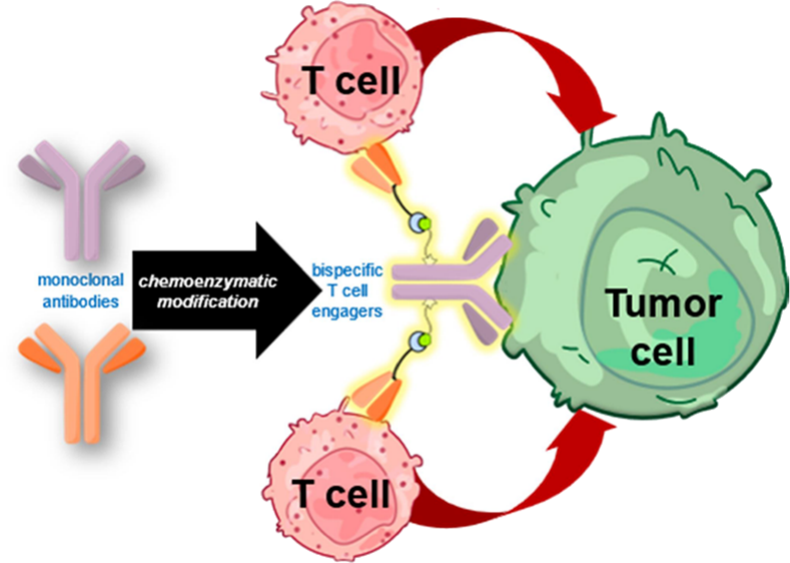

Bispecific antibodies as T cell engagers designed to display binding capabilities to both tumor-associated antigens and antigens on T cells are considered promising agents in the fight against cancer. Even though chemical strategies to develop such constructs have emerged, a method that readily converts a therapeutically applied antibody into a bispecific construct by a fully non-genetic process is not yet available. Herein, we report the application of a biogenic, tyrosine-based click reaction utilizing chemoenzymatic modifications of native IgG1 antibodies to generate a synthetic bispecific antibody construct that exhibits tumor-killing capability at picomolar concentrations. Control experiments revealed that a covalent linkage of the different components is required for the observed biological activities. In view of the highly potent nature of the constructs and the modular approach that relies on convenient synthetic methods utilizing therapeutically approved biomolecules, our method expedites the production of potent bispecific antibody constructs with tunable cell killing efficacy with significant impact on therapeutic properties.

The ability of a monoclonal antibody (mAb) to bind a specific antigen, such as a protein or epitope, on the surface of a cell has led to a variety of therapeutic applications.^1−4^ In particular, derivatives like antibody-drug conjugates (ADCs) and bispecific antibodies (bsAbs) in cancer treatment have been developed.^5−11^ Whereas ADCs are designed to deliver a toxic payload to malignant tissue,^12,13^ bsAbs bind different epitopes such as antigens on separate target cells.^14−17^ Currently, T cell or NK cell redirectors and tumor-targeted immunomodulators form the main class of bsAbs.^18^ For example, the first FDA-approved bispecific construct, blinatumomab, binds to CD19 on (malignant) B cells and CD3 on T cells, generating a cytolytic synapse that leads to lysis of the targeted cell.^19^ This success has led to the evaluation of many different formats of T cell engagers, like diabody,^20^ CrossMab,^21^ BiTE,^22^ dual affinity retargeting antibodies (DART),^23^ tandem diabody (TandAb),^24^ and more recently synthetic bispecific mAbs (SynAbs).^25^

Most bsAbs are produced via protein engineering of the native mAb framework.^26−28^ However, the evolution of bio-orthogonal (click) chemistry has facilitated the generation of novel synthetic antibody conjugates, such as ADCs.^29,30^ At the moment, strain-promoted azide alkyne cycloaddition (SPAAC)^31^ and the inverse electron-demand Diels–Alder (IEDDA) reactions between strained unsaturated carbon–carbon systems and tetrazine^32,33^ or *ortho*-quinone^34^ have been successfully applied for antibody modification.^35^

Herein, we describe the application of biogenic tyrosine-based click chemistry for the synthesis of bsAbs using native mAbs, resulting in constructs that display T-cell activation activity at picomolar concentrations.

In this approach, Fab units of the anti-CD3 antibody OKT3 were rebridged using an appropriate pyridazinedione (PD) construct to provide a single tetrazine handle per Fab, which were then connected to TCO-functionalized HER2-binding trastuzumab prepared via biogenic tyrosine-based click chemistry on the deglycosylated native mAb. As such, the correct abbreviation for this construct is rbFab-dgmAb-rbFab (in which rb = rebridged and dgmAb = deglycosylated monoclonal antibody), but we refer to these as bispecific antibody constructs (bsAcs). Contrary to most current methods, our approach enables the convenient conversion of native IgG1 antibodies into a 2:2 bsAc that combines two antigen-binding sites for each of the two different targets via a few chemical and chemoenzymatic steps.

## Results and Discussion

### BsAc Synthesis

**Scheme 1:**
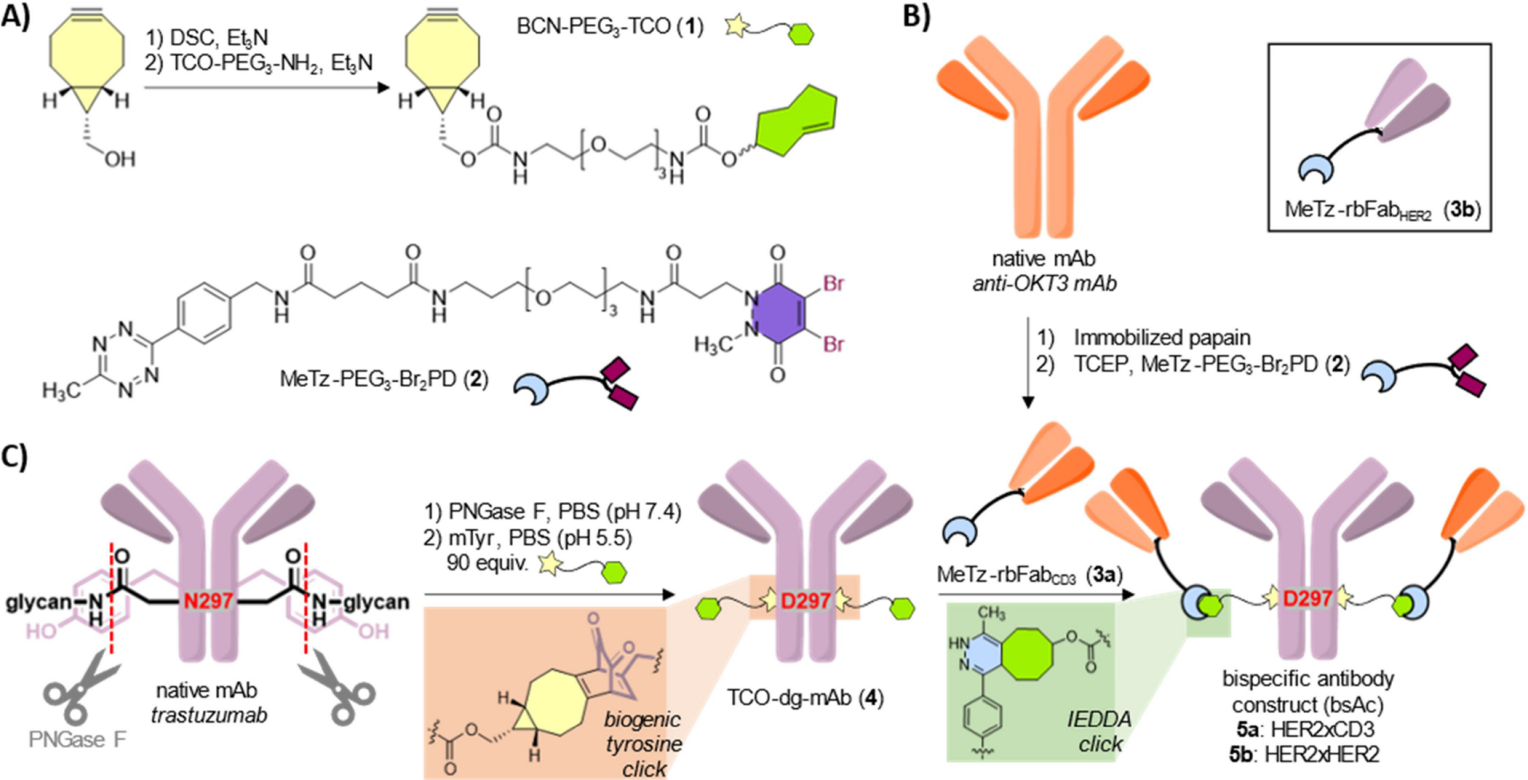
Conversion of Native mAbs to Bispecific Constructs (A) Synthesis of BCN-PEG_3_-TCO (**1**) and chemical structure of MeTz-PEG_3_-Br_2_PD (**2**). (B) Installation of MeTz handle on OKT3 Fab by rebridging the light-chain and heavy-chain Fab fragments obtained after digestion and reduction. Structure of MeTz-rbFab_HER2_ (**3b**) that is used to construct the negative control is shown in the inset. (C) Chemoenzymatic functionalization of native mAb trastuzumab with a TCO handle using BCN-PEG_3_-TCO (**1**), resulting in deglycosylated TCO-functionalized trastuzumab (**4**). The bispecific antibody construct (bsAc, **5a**) was obtained after reaction with MeTz-rbFab_CD3_ (**3a**) by TCO-Tz IEDDA.

To gain synthetic access to bsAcs from native mAbs, we first prepared appropriate BCN-PEG_3_-TCO (**1**) and MeTz-PEG_3_-Br_2_PD (**2**)^36^ linkers using convenient established procedures (Scheme 1A and Supporting Information). For the preparation of MeTz-functionalized Fab_CD3_ (**3a**, Scheme 1B), targeted digestion of the hinge region of the anti-CD3 OKT3 mAb by treatment with immobilized papain resulted in two Fab units that could be isolated from the Fc unit using protein A purification. Reduction of the C-terminally positioned intrachain disulfide bond of the obtained Fab_CD3_ fragments with an excess of TCEP enabled rebridging with the dibromopyridazinedione-based tetrazine-functionalized construct, MeTz-PEG_3_-Br_2_PD (**2**), to yield MeTz-rbFab_CD3_ (**3a**). Similarly, rebridged HER2-binding Fabs were also prepared to be incorporated in our negative control construct (i.e., MeTz-rbFab_HER2_ (**3b**), see insert Scheme 1B). An additional digestion step using immobilized pepsin was necessary to isolate the corresponding HER2-binding Fab fragments this time.^37^

Benefiting from the 150-fold higher reactivity of BCN with an *ortho*-quinone when compared to TCO,^38^ we were able to functionalize the Fc domain of trastuzumab with two TCO handles by subsequent treatment of the mAb with peptide-N-glycosidase F (PNGase F) and mushroom tyrosinase (mTyr) in the presence of BCN-PEG_3_-TCO (**1**) (Scheme 1C). As this IEDDA-compatible click handle is generated from the proteinogenic amino acid residue tyrosine, we refer to this approach as “biogenic click chemistry”. Purification by protein A column chromatography afforded the desired TCO-functionalized mAb (**4**).

**Figure 1:**
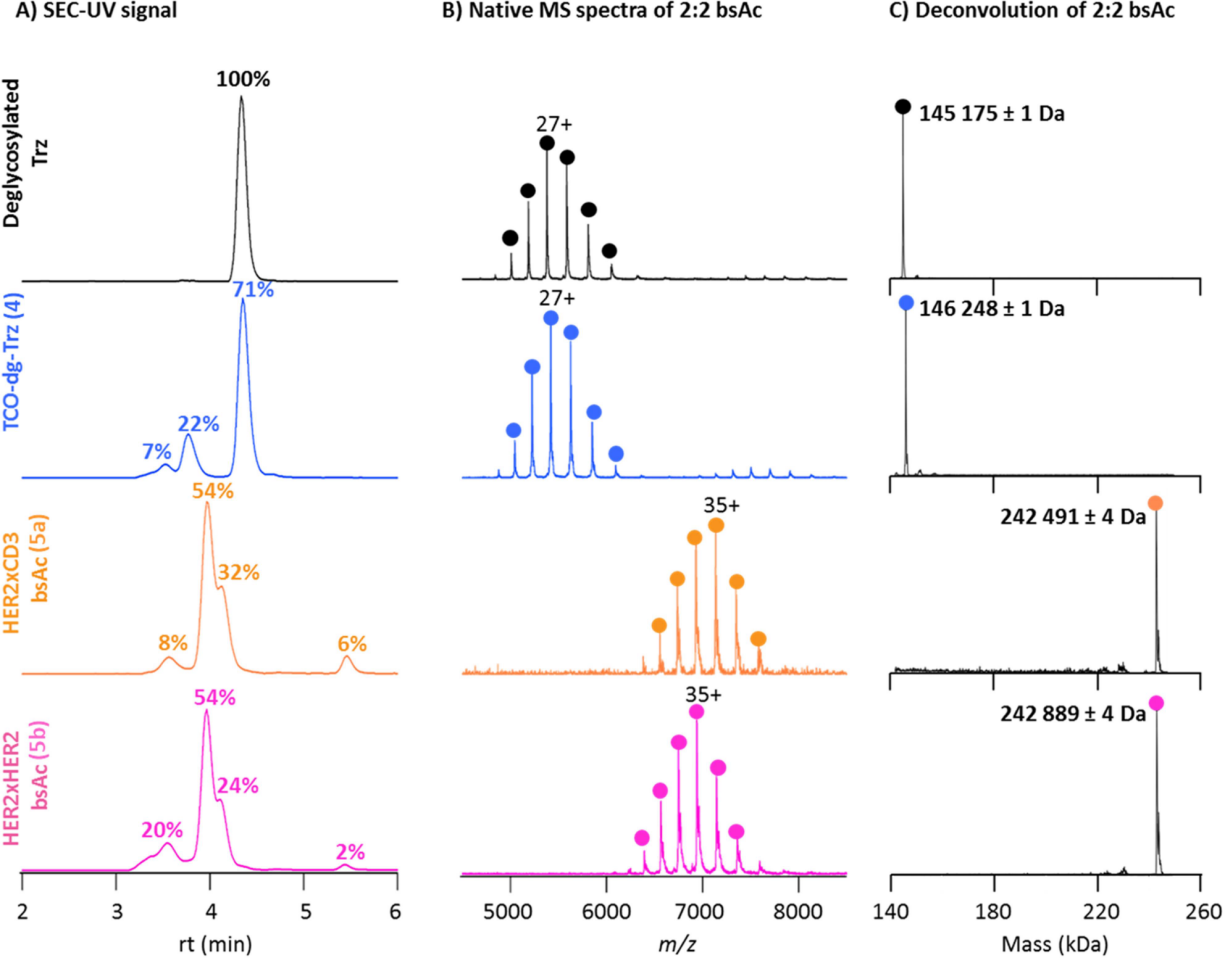
SEC-nMS analysis of trastuzumab samples obtained during assembly of the bispecific constructs via biogenic tyrosine click chemistry. (A) SEC-UV chromatogram of deglycosylated Trz (black), trastuzumab-TCO **4** (blue), HER2xCD3 bsAc **5a** (orange), and HER2xHER2 bsAc **5b** (pink). Relative quantification of each species is performed upon the integration of chromatographic peak areas. (B) Native MS spectra of the major peak from each sample namely, monomer of deglycosylated Trz (black), monomer of trastuzumab-TCO (**4**) (blue), 2:2 HER2xCD3 bsAc **5a** (orange), and 2:2 HER2xHER2 bsAc **5b** (pink). (C) Deconvoluted values of the relevant species are provided with a standard deviation obtained from at least four different charge states; masses of minor species are summarized in Supporting Information (Figure S19).

The two MeTz-functionalized Fabs, i.e., MeTz-rbFab_CD3_ (**3a**) and MeTz-rbFab_HER2_ (**3b**), were subjected to tetrazine-trans-cyclooctene IEDDA conjugation with TCO-functionalized trastuzumab (**4**) at 4 °C for 2 h in PBS of pH 7.4 (Scheme 1C). Non-reducing SDS-PAGE analysis revealed formation of the desired constructs (see Supporting Information, Figures S10 and S11), which were purified by subsequent filtration over a protein A column and size exclusion chromatography to yield the desired 2:2 HER2xCD3 bsAc (**5a**) as well as the tetravalent HER2-binding negative control (**5b**). We found that isolation of the prepared bispecific constructs was challenging (see Supporting Information, S10 and S11). In fact, analysis of the most pure fractions by native SEC-MS, which separates molecules as a function of their hydrodynamic volume and is particularly well adapted to separate, identify, and relatively quantify the different species generated during the formation of both tetrameric constructs, **5a** and **5b** (Figure 1), revealed that the reaction of TCO-deglycosylated-Trz with the different Fab subunits (either anti-CD3 or anti-HER2) resulted in earlier elution of the main peak in the chromatogram dimension (from 4.5 to 4.0 min). As expected, the hydrodynamic volume increases due to conjugation of the mAb with the Fab domains. Closer inspection showed that the main peaks (at 3.9 min) correspond to the formation of the tetravalent mAb constructs (2:2 bsAc), but that a shoulder centered at 4.1 min corresponding to the trivalent formats of the mAbs (2:1 bsAc) was also observed. Gaussian fitting of the partially coeluting species yielded relative intensities of bsAc **5a** and **5b** of 54% in both cases. For the trivalent species that are also formed, i.e., the 2:1 HER2xCD3 as a side-product of **5a** and the 2:1 HER2xHER2 as a side-product of **5b**, relative intensities of 32 and 24% were calculated, respectively. Therefore, even though full purification of these constructs was not possible, the obtained fractions mostly contained the targeted constructs and similar amounts of 2:1 byproduct.

### Biological Evaluation

**Figure 2:**
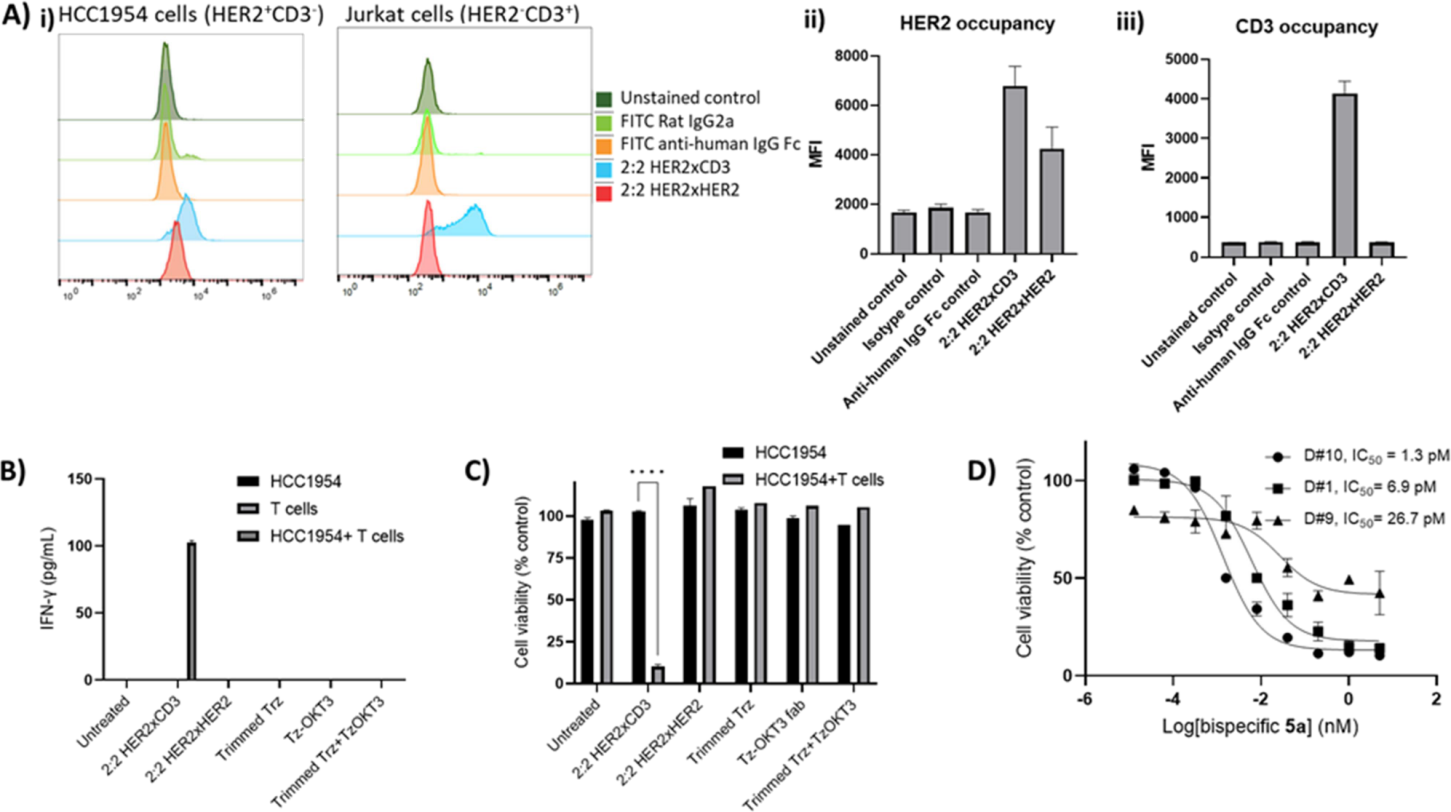
Biological activity studies of the bispecific antibody construct and controls. (A) (i) Flow cytometry analysis of binding of the various constructs to HCC1954 (HER2^+^CD3^–^) and Jurkat (HER2^–^CD3^+^) cells; (ii) binding of the constructs to HCC1954 (HER2^+^CD3^–^) cells shown as mean fluorescence intensity (MFI); and (iii) binding of the constructs to Jurkat (HER2^–^CD3^+^) cells shown as MFI (*n* = 3). (B) Induction of IFN-γ production and excretion by the various constructs and controls (5 nM) in T cells or HCC1954 (HER2^+^CD3^–^) cells alone or HCC1954/T-cell cocultures (ratio 1:10). Culture supernatant IFN-γ was quantified by ELISA at 48 h following treatment. (C) Cellular metabolism assay as a measure of HCC1954 cell viability affected by the synthetic constructs in the presence of HCC1954 (HER2^+^CD3^–^) cells alone or HCC1954/T-cell cocultures (E/T ratio 10:1, 5 nM construct). HCC1954 viability was assessed by Cell Titer-Glo at 48 h following treatment. (D) Cytotoxicity dose–response curve of the bsAc **5a** on HCC1954/T-cell cocultures (ratio 1:10) were incubated with varying concentrations (serial dilutions ranging from 0.0128 pM to 5 nM; donors are indicated with D#1, D#9, and D#10). HCC1954 cell viability was assessed by Cell Titer-Glo at 48 h following treatment, where the IC_50_ value was extrapolated. Statistical analysis was performed in GraphPad Prism (v9.5.1), where the data is presented as mean ± SEM. Statistical significance was established by Two-way ANOVA and Šídák’s multiple comparisons test (ns denotes “no significance” and **** *p* ≤ 0.0001).

The binding capability of the synthesized constructs, i.e., the isolated fractions of the 2:2 HER2xCD3 bsAc (**5a**) and the tetravalent HER2-binding negative control (**5b**), of which the analysis profile is shown in Figure 1, to HCC1954 (HER2^+^CD3^–^) and Jurkat (HER2^–^CD3^+^) cells was first evaluated by flow cytometry (Figure 2A). Briefly, HCC1954 cells were incubated with the synthesized constructs, and Fc-units of the bound constructs were stained using an FITC-labeled anti-IgG Fc antibody. Those samples stained with the isotype control and FITC-labeled anti-IgG Fc alone did not exhibit an increase in mean fluorescence intensity, while cells preincubated with samples containing the HER2xCD3 bsAc (**5a**) and the HER2xHER2 control (**5b**) displayed an increase in FITC mean fluorescence intensity as expected. This indicated that the synthetic constructs containing the HER2-binding element of trastuzumab, including the Fc unit, retained their binding to HER2^+^ cell lines. The lower staining levels detected for negative control **5b** can be attributed to the tetravalent nature of the construct, which saturates more binding sites than the other constructs while not offering additional binding options for the FITC-labeled anti-IgG Fc antibody. Following this, binding to CD3 receptors was assessed using a CD3^+^-immortalized human T cell lines (Jurkat). As expected only the cells incubated with bsAc **5a**, which contained the OKT3-derived fragments, showed an increase in mean fluorescence intensity, while cells preincubated with control construct **5b** did not reveal binding to these cells. Therefore, our synthetic constructs retained their expected binding capacity to their targeted cells after the rebridging and biogenic IEDDA click conjugation.

Next, we determined whether binding was also accompanied by T cell activation (Figure 2B). For this we determined interferon-γ levels as a qualitative indicator of T cell activation using an ELISA performed on the supernatants after 48 h following treatment with the fractions containing the different constructs (T cells were obtained from three healthy blood donors). Specifically, T cell/HCC1954 cocultures (effector:target ratio 10:1), HCC1954 monocultures, or T cell monocultures were incubated with 5 nM of bsAc **5a**, or any of the controls (i.e., untreated, tetravalent HER2 binder (**5b**), trimmed trastuzumab, MeTz-rbFab_CD3_ (**3a**), a 1:1 mixture of trimmed trastuzumab and MeTz-rbFab_CD3_ (**3a**)). As can be seen, the presence of bsAc **5a** in the T cell/HCC1954 coculture was required for significant interferon-γ expression. In fact, the absence of IFN-γ levels in the coculture that was treated with the 1:1 mixture of trimmed trastuzumab and Tz-OKT3 shows that both are required to be present in the same molecular construct in order to elicit T cell activation. Therefore, we conclude that T cell activation is restricted to the tumor microenvironment and not by simultaneous engagement of the antigen-binding site on the surface of the different cells.

After this, the capacity of each construct to activate T cells for the killing of the HER2^+^ tumor cells was assessed by using HCC1954 cells (Figure 2C). For this, T cells and HCC1954 as monocultures and T cell/HCC1954 cocultures (effector:target ratio 10:1) were incubated with 5 nM of each synthetic construct, and HCC1954 cell viability was measured following 48 h post treatment. To our delight, 90% cell death was observed in cocultures treated with the 2:2 HER2xCD3 bsAc (**5a**), where no significant cell death was observed for treatment with any of the other constructs. Furthermore, the isolated components separately, or a mixture of the non-linked isolated components, did not trigger T cell-mediated cell killing. This indicated that simultaneous binding of T cells and tumor cells by the same molecular construct was required for proper T cell engagement to the tumor cell. Lastly, IC_50_ values of the isolated fractions containing our synthetic 2:2 HER2xCD3 bsAc **5a** were determined using HCC1954 cells and T cells from three independent donors (effector:target ratio 10:1) (Figure 2D). Exposure of the mixture of cells to increasing concentrations of our bsAc **5a** revealed low IC_50_ values of 1.3–26.7 pM. Despite the variations between the performance of the T cells obtained from the different donors, the IC_50_ values in the low picomolar range reveal the potency of the constructs generated from approved mAbs by our biogenic tyrosine-based click chemistry approach. Realizing that the fractions contain 54% of the targeted 2:2 constructs and also significant amounts of 2:1 constructs, i.e., 32% for HER2xCD3 and 24% for HER2xHER2, the actual potency of the conjugates is likely higher than was observed in these studies.

## Conclusions

We developed a convenient and modular approach using both biogenic and artificial IEDDA click reactions to convert native antibodies into potent bispecific T cell-engaging bioconjugates. As such, our method provides convenient access to antibody constructs that display two different paratopes by using therapeutically approved monoclonal antibodies. While purification of the 2:2 construct from the 2:1 constructs, which are also formed, requires optimization, the synthesized 2:2 HER2xCD3 constructs displayed enhanced binding to cells that expressed either the HER2 or CD3 receptor. In fact, we found that T cell activation was restricted to a bioconjugate that contained both antigen-binding sites in one molecular construct. Clearly, our synthetic method that uses native antibodies does not hamper the biological activity of the parent mAbs, and the generated bispecific constructs retain the activity of both the mAbs, activating the T lymphocytes against HER2^+^ cells, inducing tumor cell death even in the very low pM range. Current studies are focused on dissecting the potency of the 2:2 constructs in relation to the 2:1 constructs and improving our synthetic method toward the preparation of different and more diverse bsAcs.

## Supporting Information Available

Synthetic chemistry experimental details, conjugation experiments and *in vitro* assays, ^1^H and ^13^C spectra, and full LC-MS spectra including TIC trace and deconvoluted spectra (PDF)
