# Supplementary material for: Rapid Access to Potent Bispecific T Cell Engagers Using Biogenic Tyrosine Click Chemistry
Source: Bioconjug Chem. 2023 Nov 14;34(12):2215–20. doi: 10.1021/acs.bioconjchem.3c00357 (PMC10739583; doi:10.1021/acs.bioconjchem.3c00357)
Supplement: Supplementary file 1 — bc3c00357_si_001.pdf [file bc3c00357_si_001.pdf]

Supporting Information

for

Rapid Access to Potent Bispecific T cell Engagers using Biogenic Tyrosine Click Chemistry

Irene Shajan,[a] Léa N.C. Rochet,[b] Shannon R. Tracey,[c] Bianka Jackowska,[c] Rania Benazza,[d] Oscar Hernandez-Alba,[d] Sarah Cianférani,[d] Christopher J. Scott,[c] Floris L. van Delft,[a,e] Vijay Chudasama,[b]* Bauke Albada[a]*

[a]Laboratory of Organic Chemistry, Wageningen University & Research, Stippeneng 4, Wageningen, 6807 WE, the Netherlands

[b]Department of Chemistry, University College London, 20 Gordon St, London, WC1H 0AJ, United Kingdom

[c]Patrick G Johnston Centre for Cancer Research, School of Medicine, Dentistry and Biomedical Sciences, Queen’s University Belfast, 97 Lisburn Road, BT9 7BL, Belfast, United Kingdom

[d]Laboratoire de Spectrométrie de Masse BioOrganique, Université de Strasbourg, CNRS, IPHC UMR 7178, 67000 F-Strasbourg, France

Infrastructure Nationale de Protéomique ProFI – FR2048, 67087 Strasbourg, France

[e]Synaffix BV – A Lonza company, Kloosterstraat 9, 5349 AB, Oss, the Netherlands

*Corresponding author: bauke.albada@wur.nl

Table of Contents

	Page
General procedures	3
Antibody modifications and bioconjugations	3
Synthesis of linkers	11
In vitro evaluation of the bispecific antibodies	14
NMR spectra	15
LC-MS data	17
SEC-nMS of the antibody conjugates	18
Literature references	19

General procedures

Starting materials, reagents, and solvents were purchased from commercial vendors and used as received unless stated otherwise. TCO-PEG_3-NH_2 was purchased from BroadPharm® (San Diego, CA, United States). *Endo*-bicyclo[6.1.0]non-4-yn-9-ol (BCN-OH) was provided by Synaffix (Oss, the Netherlands). Mushroom tyrosinase was purchased from Sigma-Aldrich. Trastuzumab (Herzuma) was obtained from the pharmacy. OKT3 antibody was purchased from BioLegend®. PNGase F was obtained from New England Biolabs (NEB). Reactions were monitored by thin-layer chromatography (TLC) using Merck aluminum sheets (Silica gel 60 F254). ^{1}H NMR and ^{13}C NMR spectra were recorded using a Brüker AV-400 (400 and 101 MHz, respectively) spectrometer in $CDCl_3$. Chemical shifts are given in ppm (δ) relative to the residual solvent peak or tetramethyl silane (0 ppm) as internal standard and coupling constants are given in Hz. High-resolution mass spectrometry (HRMS) analysis was performed with an Q-Exactive Focus Mass Spectrometer (Thermo Fisher), equipped with an electrospray ion source (ESI) in positive mode. RP-HPLC analysis was performed on an Agilent 1290 series instrument. The sample (20 µL) was injected with 0.6 mL/min onto MAbPac RP 3.0 × 100 mm 4 µm (Thermo Scientific) with a column temperature of 80 °C. A linear gradient was applied in 15 minutes from 25% to 40% acetonitrile in 0.1% FA and water.

General procedure for reducing SDS-PAGE, Coomassie staining and fluorescence detection
6% acrylamide gels were prepared according to BIO-RAD bulletin 6201 protocol. 5 µg of the antibody solution was diluted in 5 µL PBS (pH 7.4). 5 µL of 2× sample buffer was added to the sample and heated to 95 °C for 5 minutes. After loading the samples, the gel was run using a BIO-RAD Mini-PROTEAN Tetra Vertical Electrophoresis Cell at 120 volts until completion. The gel was stained using a 1 g/L Coomassie Brilliant Blue R-250 in 5:4:1 (v/v/v) methanol:water:acetic acid solution (30 minutes soak). The gel was subsequently destained using 5:4:1 (v/v/v) methanol:water:acetic acid for 30 minutes, after which it was further destained overnight using demineralized water.

General procedure for analytical RP-HPLC-MS
Prior to RP-HPLC analysis, 40 µL of 12.5 mM DTT in 100 mM Tris.HCl pH 8 was added to IgG (10 µL, 1 mg/mL in PBS pH 7.4) and incubated for 15 minutes at 37 °C. Prior to injection, the reaction was quenched by adding 49% acetonitrile, 49% water, 2% formic acid (50 µL). The mass spectrometer was operated with a spray voltage of 3.9 kV. Acquisitions were performed on the m/z range 500-3000. Deconvoluted spectra were obtained using UniDec software.[39]

Antibody modifications and bioconjugations

Enzymatic deglycosylation of trastuzumab
Trastuzumab (30 mg, 38.4 mg/mL in PBS pH 7.4) was incubated with PNGase F (37.5 µL, 18750 units) at 37 °C. After overnight incubation the antibody was dialyzed (thrice to PBS pH 5.5) and concentrated to 118.2 mg/mL. Mass spectrometric analysis of a sample after DTT treatment showed the mass corresponding to heavy chain of the expected product (observed mass 49147 Da, Fig. S1D).

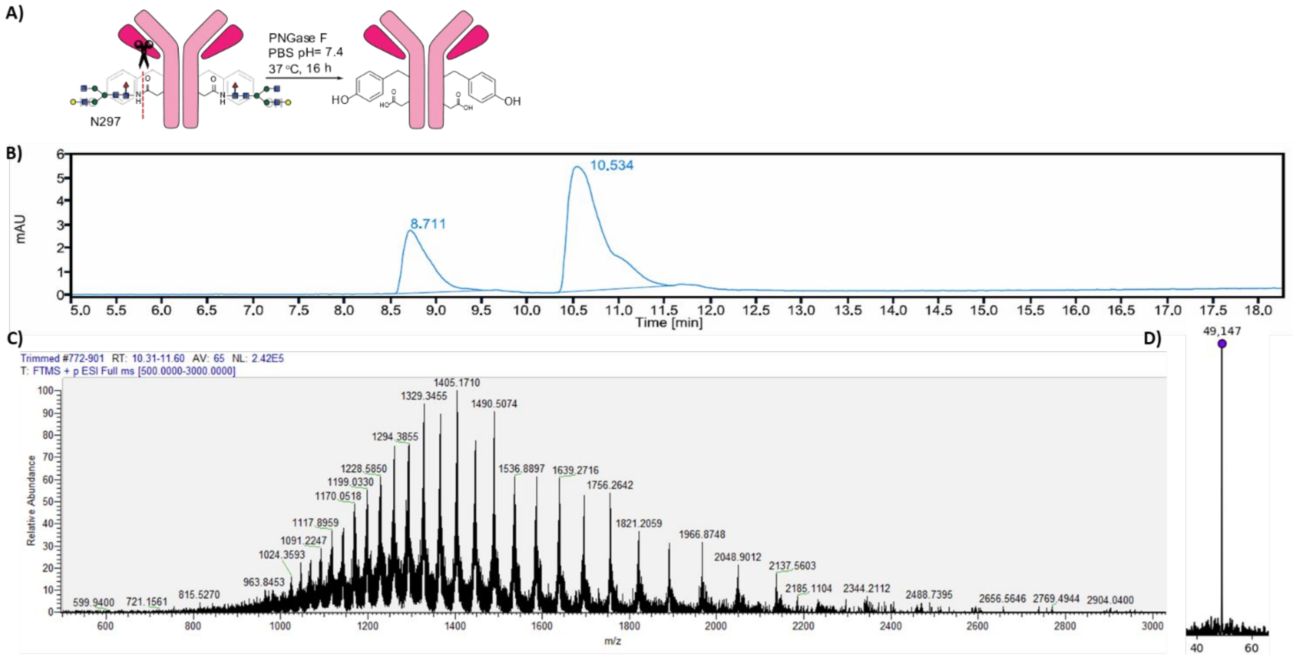

Fig. S1: A) Schematic representation of deglycosylation of trastuzumab using PNGase F. B) HPLC trace of DTT digested deglycosylated trastuzumab, with light chain at t_R 8.7 min and heavy chain at t_R 10.5 min. C) Mass spectrum of the heavy chain of deglycosylated trastuzumab. D) Deconvoluted mass spectrum of heavy chain (the peak eluting at t_R = 10.5 min in panel B).

Conjugation of deglycosylated trastuzumab with BCN-PEG$_3$-TCO (**1**)

Deglycosylated trastuzumab (13.5 µL, 147.8 mg/mL, 2 mg in PBS pH 5.5) was diluted with 840 µL PBS pH 5.5 and incubated with BCN-PEG$_3$-TCO (**1**, 12.9 µL, 50 mg/mL in DMSO) and mushroom tyrosinase (123. 9 µL, 10 mg/mL in phosphate buffer pH 6.0) at 4 °C. After overnight incubation, the product was purified using protein A purification and buffer exchanged to PBS pH 7.4. RP-LC-MS analysis of the DTT digested product was performed as described above and indicated clean conversion and showed one major heavy chain product (observed mass 49681 Da) corresponding to the expected product.

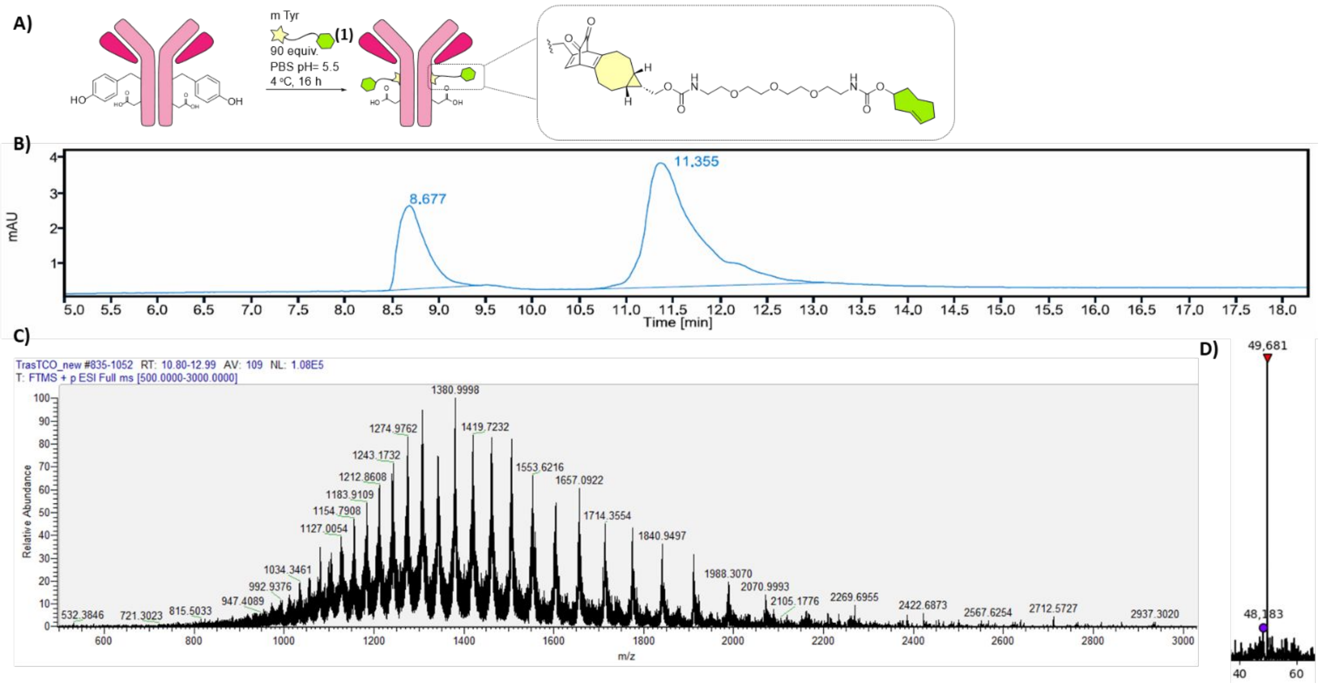

Fig. S2: A) Generation of TCO-modified trastuzumab via SPOCQ reaction. B) HPLC trace of DTT digested TCO-modified trastuzumab, with the LC at t_R 8.7 min and HC at t_R 11.4 min. C) Mass spectrum of heavy chain of TCO-modified trastuzumab. D) Deconvoluted mass spectrum of heavy chain that eluted at t_R 11.4 min (Fig. S2B).

Digestion of mAb to Fab$_{CD3}$ fragment (OKT3)

Anti-CD3 antibody was buffer exchanged into digest buffer (20 mM NaH_2PO_4, 20 mM, pH 7.0, 10 mM EDTA, 80 mM cysteine.HCl). Immobilized papain (1.56 mL, 0.1 mL/ 1 mg mAb) was washed three times with digest buffer. The mAb solution (1 mL, 107 µM) was then added to the immobilized papain and the heterogeneous mixture was incubated under constant agitation (1100 rpm) for 5 h at 37 °C. The resin was separated from the digest using a filter column and washed three times with Pierce^{TM} protein A binding buffer. The digest was combined with the washes and buffer-exchanged completely in Pierce^{TM} Protein A binding buffer. The volume was adjusted to 2 mL and the Fab and Fc fragments were separated using Protein A purification, following the manufacturer's protocol. Yield: 83%.

Digestion of mAb to Fab$_{HER2}$ fragment (Trastuzumab)

Anti-HER2 antibody was buffer exchanged into sodium pepsin digest buffer (20 mM NaOAc, pH 3.1). Immobilized pepsin (732 µL) was washed 4 times with pepsin digest buffer and the mAb solution (1 mL, 107 µM) was added to this. The mixture was incubated for 5 h at 37 °C under constant agitation (1100 rpm). The resin was separated from the digest using a filter column and washed 3 times with papain digest buffer (50 mM sodium phosphate, 150 mM NaCl, 1 mM EDTA, pH 6.8). The digest (F(ab')$_2$ solution) was combined with the washes and the volume adjusted to 0.5 mL. Immobilized papain (1.22 mL, 0.25 mg/mL) was activated with 10 mM DTT (in papain digest buffer) with constant agitation (1100 rpm) for 90 min at 37 °C. The resin was washed 4 times with papain digest buffer (without DTT) and the 0.5 mL of F(ab')$_2$ solution was added. The mixture was incubated for 24 h at 37 °C under constant agitation (1100 rpm). The resin was separated from the digest using a filter column, washed 3 times with PBS and the digest combined with the washes. The buffer was exchanged completely for PBS (10 mM phosphate, 2.7 mM KCl, 137 mM NaCl, pH 7.4), and the volume adjusted to 0.5 mL. Yield: 67%.

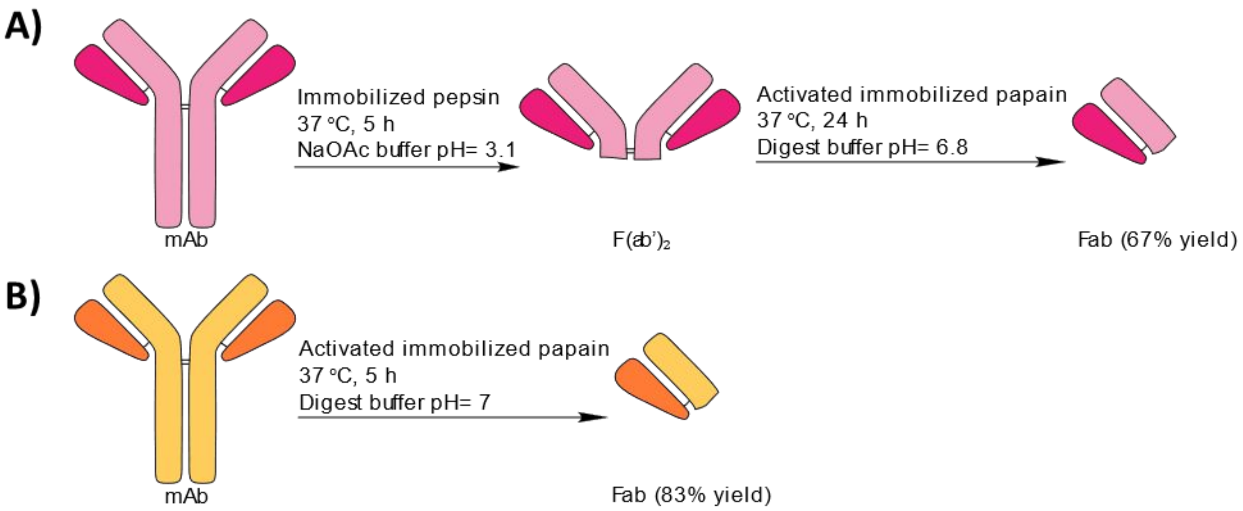

Fig. S3: Preparation of the A) Fab_{HER2} and B) Fab_{CD3} fragment from their respective parent antibodies.

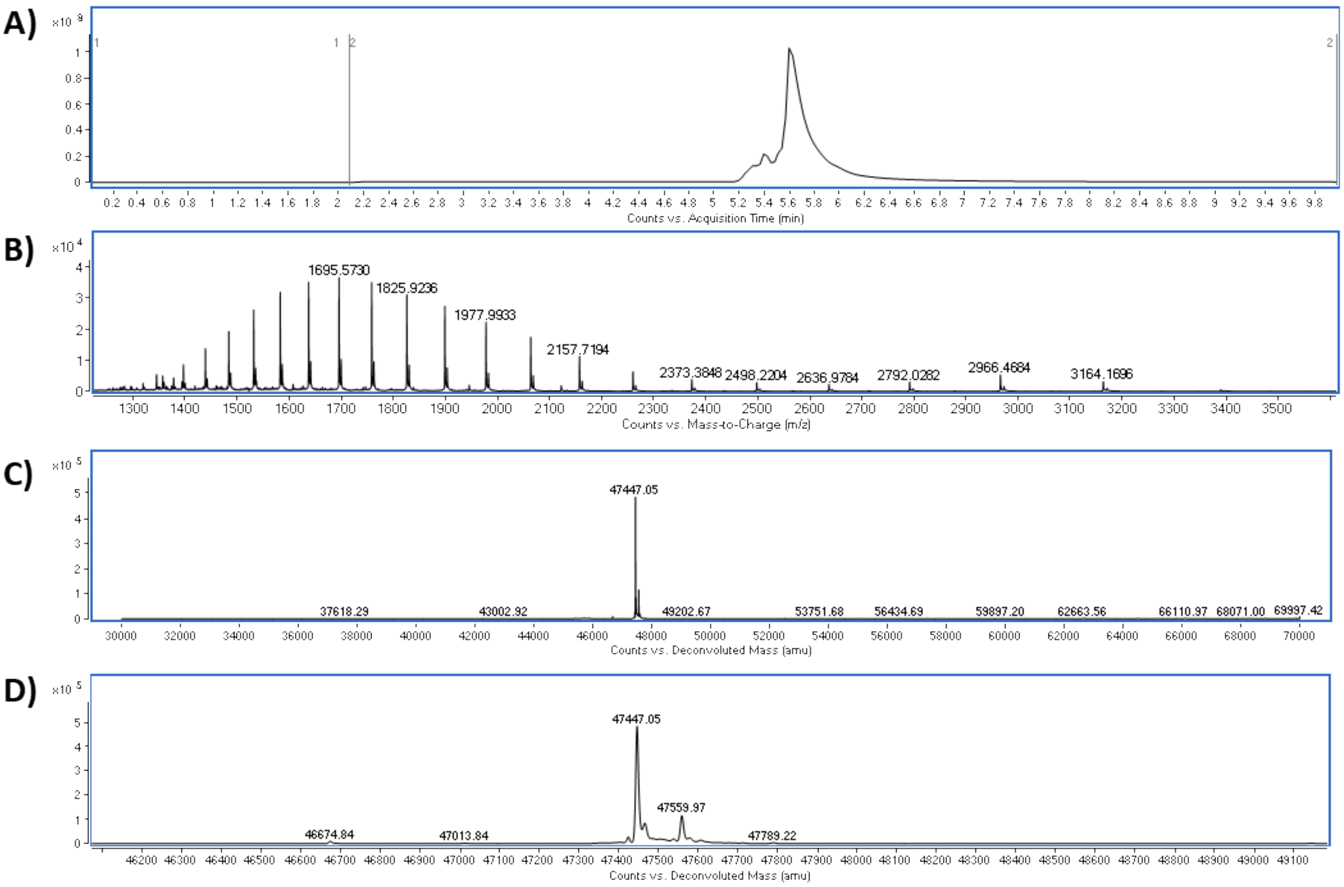

Fig. S4: A) MS chromatogram, B) mass spectrum, C) deconvoluted spectrum and D) zoomed deconvoluted spectrum of Fab_{CD3}. Expected masses: 47447, 47560 Da.

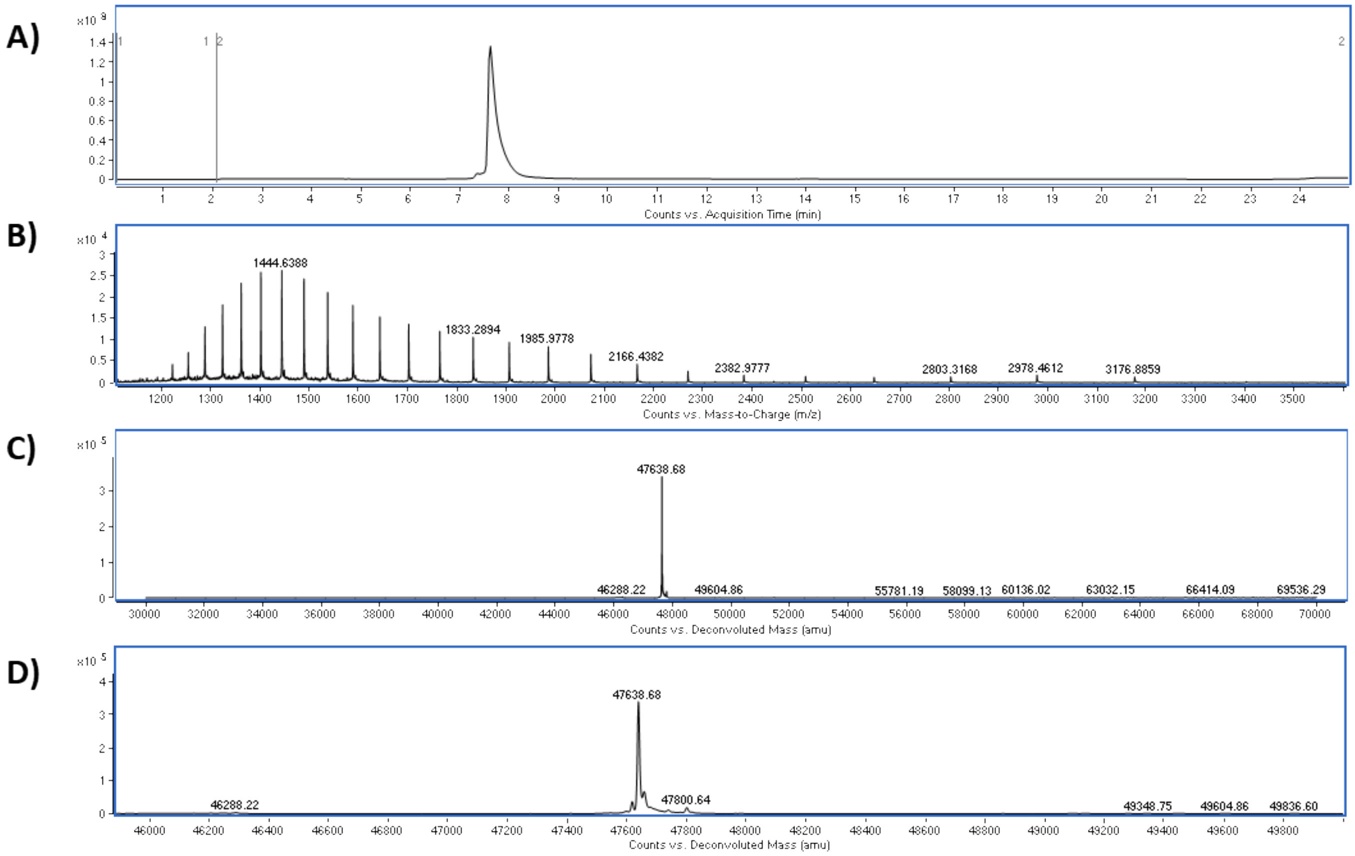

Fig. S5: A) MS chromatogram, B) mass spectrum, C) deconvoluted spectrum and D) zoomed deconvoluted spectrum of Fab$_{tHER2}$. Expected mass: 47639 Da.

Reduction of Fab fragment and subsequent Fab re-bridging with tetrazine-dibromopyridazinedione

A solution of 20 mM TCEP was prepared by dissolving TCEP-HCl (15 mg) in 5 × BBS (2.6 mL). Fab (45 µM, 1700 µL) was prepared in BBS and BBS 5 × (600 and 500 µL respectively) followed by addition of 20-60 equivalents of TCEP (20 mM in 5 × BBS, 77-230 µL; 60 eq. for Fab_{CD3} and 20 eq. for Fab_{HER2}). The mixture was incubated for 120 min at 37 °C under constant agitation (300 rpm). Excess TCEP was removed using Vivaspin 500 10,000 MWCO and Zeba^{TM} Spin 7K MWCO washes in BBS EDTA (Re-bridging was carried out by adding 10 equivalents of pyridazinedione Br_2PD-Tz to the solution of reduced Fab in BBS EDTA (38 µL, 20 mM in DMSO) and the mixture incubated at 37 °C with constant agitation (300 rpm) over 2 h. Excess PD was removed using Vivaspin 500 10,000 MWCO and Zeba^{TM} Spin 7K MWCO washes in BBS. The purity and identity of the sample was assessed by non-reducing SDS-PAGE and (HR)-LC-MS. Yields: 78% for MeTz-Fab_{CD3} (**4a**) and 72% for MeTz-Fab_{HER2} (**4b**).

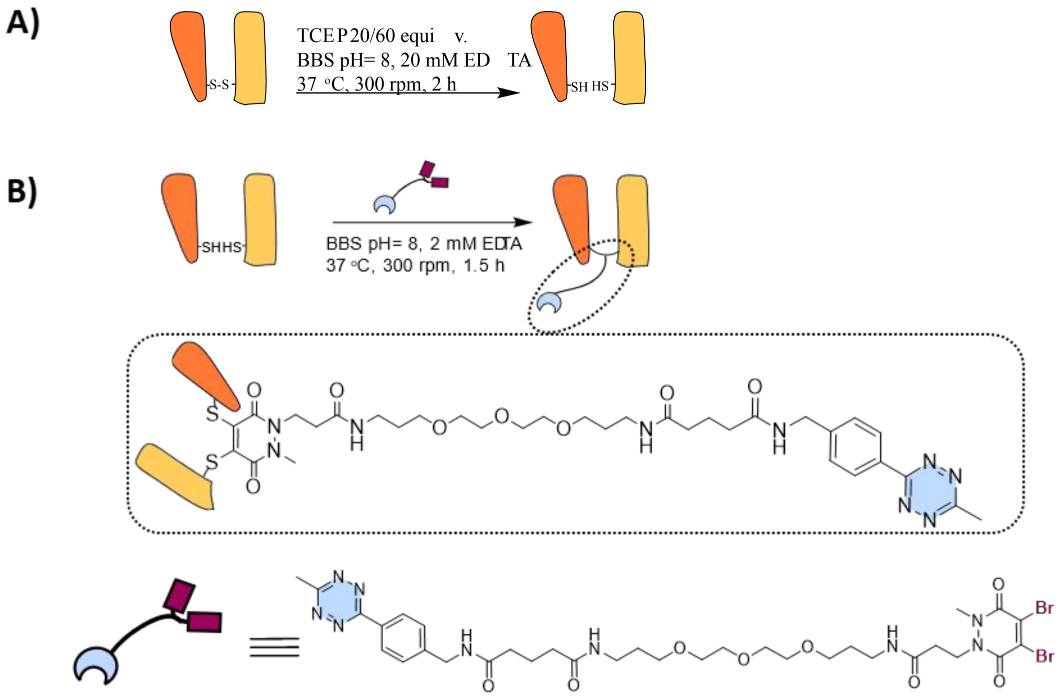

Fig. S6: A) Reduction of the Fab fragment, and B) Fab re-bridging using tetrazine-dibromopyridazinedione (**2**).

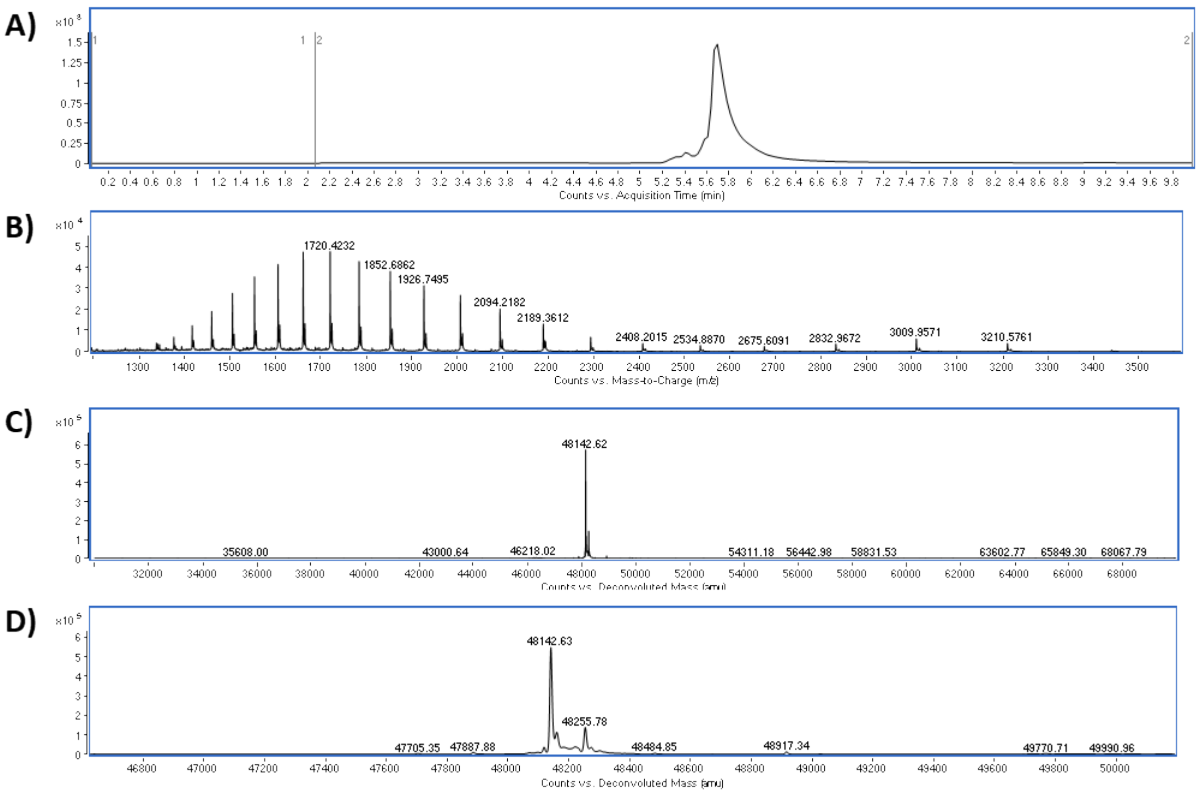

Fig. S7: A) MS chromatogram, B) mass spectrum, C) deconvoluted spectra and D) zoomed deconvoluted spectra of MeTz-rbFab$_{CD3}$ (**3a**). Expected masses: 48142, 48255 Da.

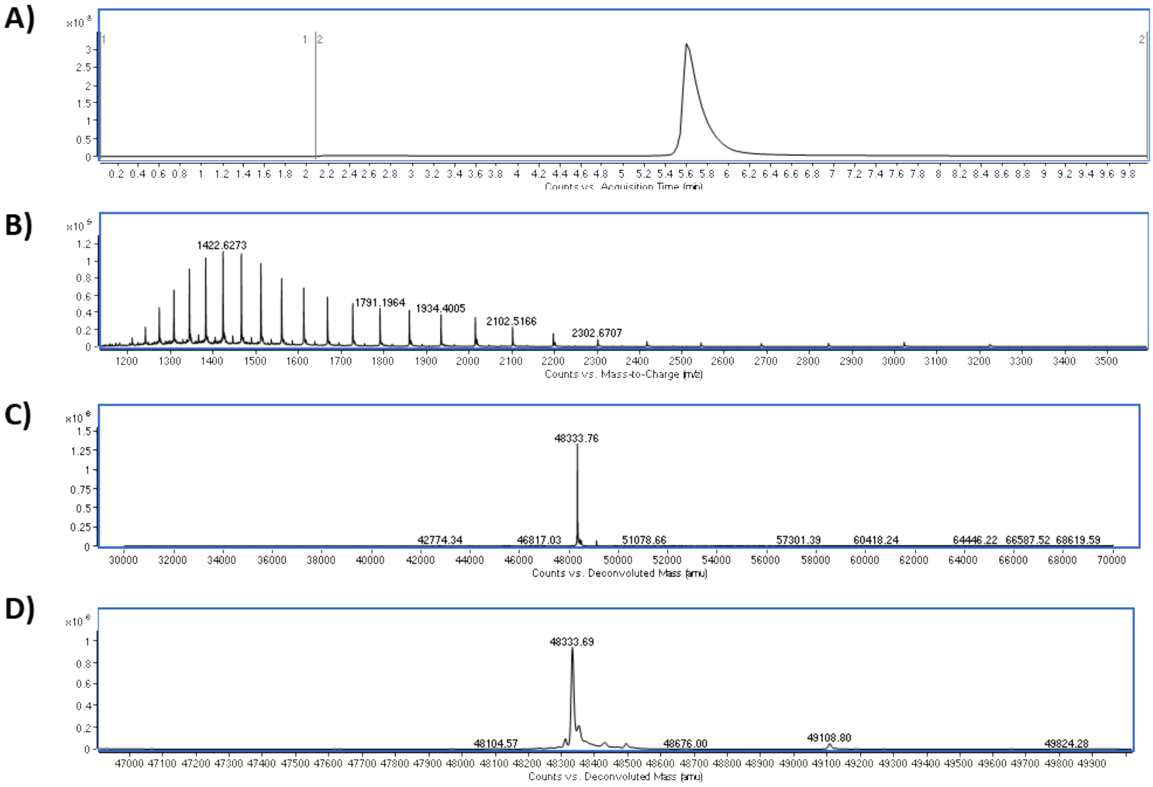

Fig. S8: A) MS chromatogram, B) mass spectrum, C) deconvoluted spectra and D) zoomed deconvoluted spectra of MeTz-rbFab$_{HER2}$ (**3b**). Expected masses: 48334 Da.

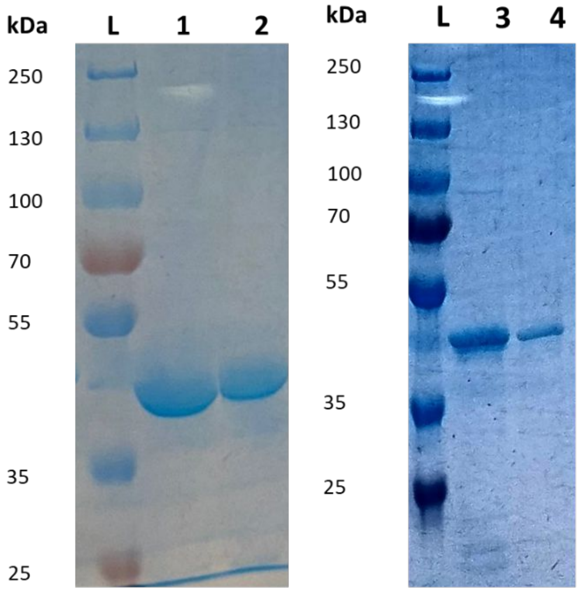

Fig. S9: 12% non-reducing SDS-PAGE gel of digested Fabs and rebridged Fabs. **L:** ladder, **lane 1**: Fab$_{CD3}$, **lane 2**: MeTz-rbFab$_{CD3}$ (**3a**), **lane 3**: Fab$_{HER2}$, **lane 4**: MeTz-rbFab$_{HER2}$ (**3b**).

Generation of 2:2 HER2xCD3 bispecific antibody **5a**

TCO-modified trastuzumab (**4**) (158.4 µL, 6.3 mg/mL, 1 mg in PBS pH 7.4) was incubated with MeTz-rbFab$_{CD3}$ (**3a**) (35.8 µL, 55.4 mg/mL, 2.0 mg in PBS pH 7.4) at 4 °C for 2 h. The product (**5a**) was purified using protein A purification followed by size-exclusion chromatography, and analyzed using non-reducing SDS-PAGE and native SEC-nMS.

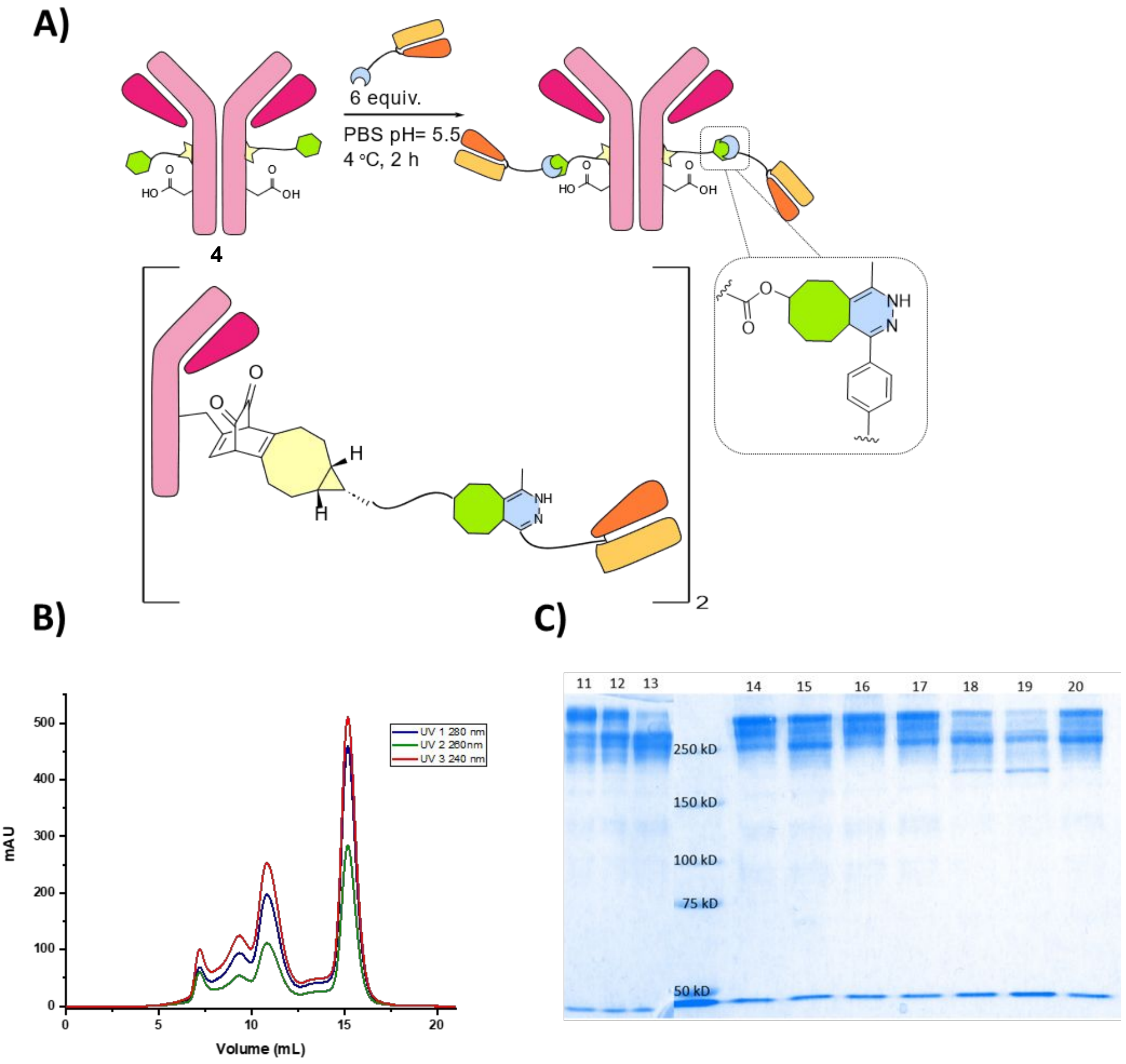

Fig. S10: A) Generation of 2:2 HER2xCD3 bispecific antibody via TCO-tetrazine IEDDA reaction, B) SEC purification of the reaction mixture, and C) 6% non-reducing SDS-PAGE of the SEC fractions.

Generation of the HER2xHER2 antibody construct **5b**

TCO-modified trastuzumab **4** (158.4 µL, 6.3 mg/mL, 1 mg in PBS pH 7.4) was incubated with MeTz-rbFab$_{HER2}$ (**3b**) (38.8 µL, 51.1 mg/mL, 2.0 mg in PBS pH 7.4) at 4 °C for 2 h. The product (**5b**) was purified using protein A purification followed by size-exclusion chromatography, and analyzed using non-reducing SDS-PAGE and native SEC-nMS.

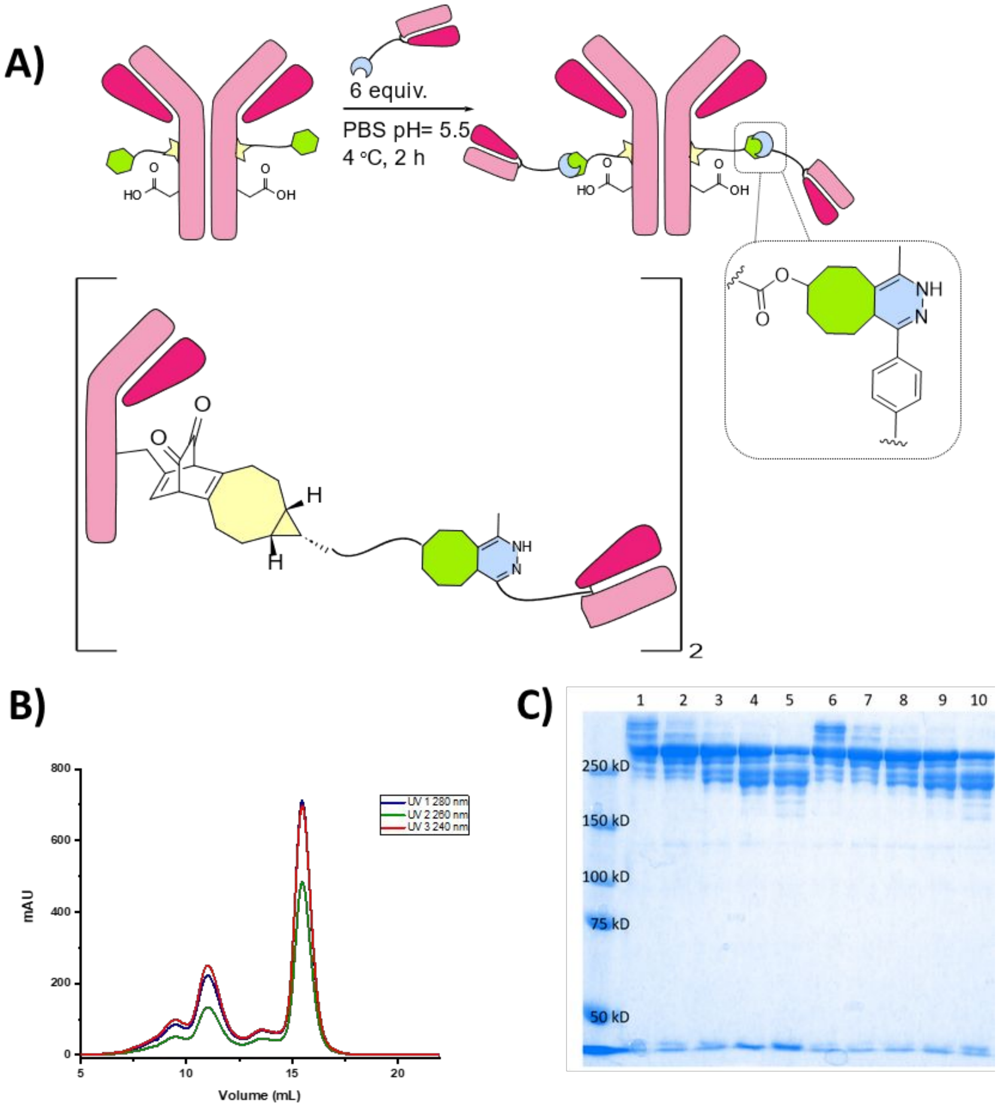

Fig. S11: A) Generation of 2:2 HER2xHER2 bispecific antibody via TCO-tetrazine IEDDA reaction, B) SEC purification of the reaction mixture, and C) 6% non-reducing SDS-PAGE of the SEC fractions.

Synthesis of Linkers

The following section describes the synthesis of the BCN-PEG$_3$-TCO linker that was installed on trastuzumab.

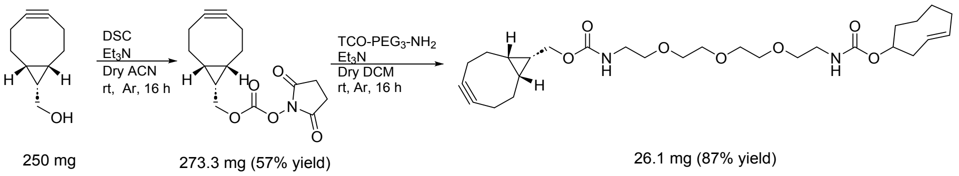

Fig. S12: Synthesis of BCN-PEG$_3$-TCO (**1**).

BCN-OSu

To a solution of BCN-OH (250 mg) in 6 mL ACN were added disuccinimidyl carbonate (DSC, 853 mg, 2 equiv.) and triethylamine (695.8 µL, 3 equiv.). After the mixture was stirred for 2 h, it was concentrated *in vacuo*. The residue was purified by silica gel column chromatography (25 → 50% EtOAc in hexane) to afford the product as a white solid (273.3 mg, 57% yield). ^{1}H NMR (400 MHz, $CDCl_3$): δ 4.38 (d, 2H, *J*= 8 Hz), 2.77 (s, 4H), 2.29–2.13 (6H, m), 1.54–1.39 (3H, m), 1.04–0.94 (2H, m). ^{13}C NMR (101 MHz, $CDCl_3$): δ 168.7, 151.6, 98.7, 70.3, 29.0, 25.5, 21.5, 20.7, 17.2.

BCN-PEG$_3$-TCO (**1**)

TCO-PEG$_3$-NH_2 (19.7 mg) was dissolved in 2 mL dry DCM. To this, 16.2 µL triethylamine followed by BCN-OSu (16.7 mg, 1.1 equiv.) were added. After stirring at room temperature for 2 h under argon atmosphere, the reaction mixture was evaporated and purified via silica gel chromatography (1% MeOH-DCM) to afford the product as a colorless oil (26 mg, 87% yield). ^{1}H NMR (400 MHz, $CDCl_3$): δ 5.5–5.43 (1H, m), 5.21–4.93 (1H, bs), 4.29–2.25 (0.62H, bs), 4.90 (2H, d, *J*= 8 Hz), 3.59–3.55 (8H, m), 3.51–3.46 (4H, m), 3.31–3.29 (4H, m), 2.30–2.07 (9H, m), 1.94–1.82 (3H, m), 1.68–1.47 (6.7H, m), 1.48–1.27 (1.48H, m), 1.19 (0.81H, m), 0.90–0.85 (2H, m). ^{13}C NMR (101 MHz, $CDCl_3$): δ 156.8, 156.3, 132.9, 133.0, 129.3, 127.3, 98.8, 70.5, 70.3, 70.3, 70.2, 70.2, 41.2, 38.7, 34.3, 32.5, 31.0, 29.1, 21.4, 20.1, 17.8. ESI-MS (observed m/z): 521.3132 Da.

Synthesis of dibromopyridazinedione-tetrazine (Br$_2$PD-Tz) (**2**)

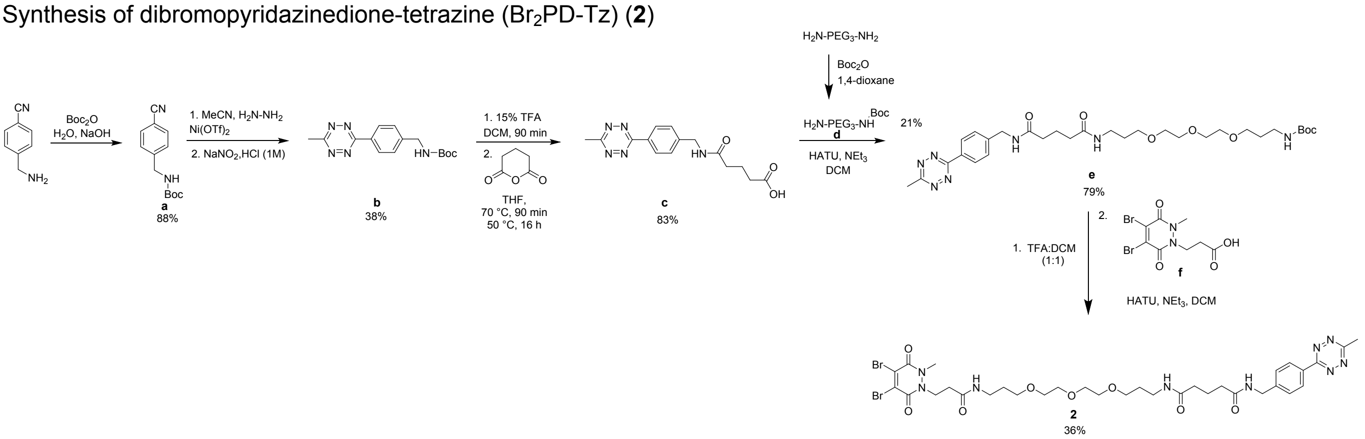

Fig. S13: Synthesis scheme for dibromopyridazinedione-tetrazine (**2**)

tert-butyl (4-cyanobenzyl)carbamate (**a**)[40]

To a stirring solution of NaOH (3.6 g, 89.1 mmol) and di-*tert*-butyl dicarbonate (7.1 g, 32.6 mmol) in H_2O (30 mL) was added, at room temperature, a pre-dissolved solution of 4-(aminomethyl)benzonitrile (5.0 g, 29.7 mmol) in H_2O (30 mL). The mixture was stirred for 16 h, after which time a white precipitate had formed. The mixture was then filtered, the solid washed with H_2O (100 mL), and the resulting solid dried under vacuum to yield compound **a** as a white solid (6.09 g, 26.2 mmol, 88%). ^{1}H NMR (400 MHz, CDCl3) δ 7.62 (d, J = 8.3, 2H), 7.38 (d, J = 8.3, 2H), 4.96 (br s, 1H), 4.37 (d, J = 5.9 Hz, 2H), 1.46 (s, 9H); ^{13}C NMR (100 MHz, CDCl3) δ 144.7 (C), 132.5 (C), 127.9 (C), 118.8 (C), 111.1 (C), 80.1 (C), 44.3 (CH2), 28.4 (CH3); IR (solid) 3350, 2974, 2927, 2226, 1692 cm^{-1}.

tert-butyl (4-(6-methyl-1,2,4,5-tetrazin-3-yl)benzyl)carbamate (**b**)[41]

To a stirring suspension of *tert*-butyl carbamate **a** (1.0 g, 4.3 mmol) in acetonitrile (10.5 mL, 43 mmol) were added Ni(OTf)$_2$ (768 mg, 2.2 mmol) and hydrazine hydrate (80% w/w, 10.5 mL, 215 mmol). The reaction was heated to 65 °C and stirred for 20 h. After this time, the reaction was cooled to room temperature and a solution of $NaNO_2$ (35.6 g, 516 mmol) in H_2O (258 mL) was added slowly to the reaction mixture. This was followed by addition of 1 M HCl until no more gas is formed, during which time the reaction turned dark purple. The reaction was then extracted with EtOAc (4×200 mL), dried ($MgSO_4$), filtered and the solvent was then removed in vacuo. The resulting residue was purified by flash column chromatography (0% to 5% Et_2O/DCM) to yield tetrazine **b** as a pink solid (497 g, 1.65 mmol, 38%). ^{1}H NMR (400 MHz, $CDCl_3$) δ 8.55 (d, J = 8.4 Hz, 2H), 7.50 (d, J = 8.3 Hz, 2H), 4.97 (br s, 1H), 4.44 (d, J = 5.8 Hz, 2H), 3.09 (s, 3H), 1.48 (s, 9H); ^{13}C NMR (100 MHz, $CDCl_3$) δ 167.3 (C), 164.0 (C), 144.0 (C), 130.1 (C), 128.3 (C), 128.1 (C), 80.1 (C), 28.5 (CH2), 21.1 (CH3); IR (solid) 3339, 2974, 2928, 1696, 1516 cm^{-1}.

5-((4-(6-methyl-1,2,4,5-tetrazin-3-yl)benzyl)amino)-5-oxopentanoic acid (**c**)[42]

tert-Butyl (4-(6-methyl-1,2,4,5-tetrazin-3-yl)benzyl)carbamate **b** (400 mg, 1.33 mmol) was dissolved in a mixture of TFA and DCM (1:4, 10 mL) and the solution was stirred at room temperature for 2 h. The solvent was then removed in vacuo and the mixture re-dissolved in THF (50 mL). To this solution was added glutaric anhydride (174 mg, 2.66 mmol) and the mixture stirred at 55 °C for 16 h. The solvent was removed in vacuo and the mixture re-dissolved in sat. aq. K_2CO_3 solution (50 mL). The mixture was then acidified with 15% HCl aq. solution until the mixture stopped producing CO_2 (g) on addition of acid. The mixture was then extracted with EtOAc (3 × 50 mL) and the combined organic phases washed with H_2O (4 × 30 mL) and brine (30 mL), and then dried ($MgSO_4$). Any precipitate formed during extraction was re-dissolved in sat. aq. K_2CO_3 solution (30 mL) and the work-up was repeated on this solution and the dried organic phases were combined, filtered and the solvent removed in vacuo to yield compound **c** as a purple powder (350 mg, 1.1 mmol, 83%) without further purification. ^{1}H NMR (400 MHz, $CDCl_3$) δ 8.41 (d, J = 8.4, 2H), 7.51 (d, J = 8.5 Hz, 2H), 4.38 (d, J = 6.0 Hz, 2H), 2.98 (s, 3H), 4.27 (q, J = 7.4 Hz, 4H), 1.76 (quint., J = 7.4 Hz, 2 H); ^{13}C NMR (100 MHz, $CDCl_3$) δ 174.2 (C), 171.9 (C), 167.1 (C), 163.2 (C), 144.5 (C), 130.4 (C), 128.0 (C), 127.5 (C), 41.9 (CH2), 34.4 (CH2), 33.0 (CH2), 20.8 (CH3), 20.7 (CH2); IR (solid) 3271, 3025, 2973, 2923, 2880, 1694, 1630, 1523 cm^{-1}.

tert-Butyl (3-(2-(2-(3-aminopropoxy)ethoxy)ethoxy)propyl)carbamate (**d**)[43]

To a solution of 3,3'-((oxybis(ethane-2,1-diyl))bis(oxy))bis(propan-1-amine) (5.0 g, 22.7 mmol) in 1,4-dioxane (40 mL) was added dropwise di-*tert*-butyl dicarbonate (618 mg, 2.83 mmol, pre-dissolved in 1,4-dioxane (15 mL)) over 2 h, ensuring that the

temperature did not exceed 21°C. After this time, the reaction mixture was stirred at 21 °C for a further 1 h. Following this, the reaction mixture was concentrated *in vacuo*, the crude residue dissolved in H_2O (50 mL), and the organic layer extracted with EtOAc (4 × 30 mL). The organic layers were combined, dried ($MgSO_4$) and concentrated in vacuo to yield *tert*-butyl (3-(2-(2-(3-aminopropoxy)ethoxy)ethoxy)propyl)carbamate **d** (1.40 g, 4.82 mmol, 21%) as a colorless oil. ^{1}H NMR (600 MHz, $CDCl_3$) δ 5.10 (br s, 1H), 3.63-3.54 (m,12H), 3.22-3.21 (m, 2H), 2.83 (t, J = 6.7 Hz, 2H), 1.76-1.73 (m, 4H), 1.42 (s, 9H); ^{13}C NMR (150 MHz, $CDCl_3$) δ 156.3 (C), 79.0 (C), 70.7 (CH2), 70.7 (CH2), 70.3 (CH2), 70.3 (CH2), 69.7 (CH2), 69.6 (CH2), 39.8 (CH2), 38.6 (CH2), 32.8 (CH2), 29.8 (CH2), 28.6 (CH3); IR (thin film) 3360, 2928, 2865, 1696, 1521, 1102 cm^{-1}

.

tert-Butyl (1-(4-(6-methyl-1,2,4,5-tetrazin-3-yl)phenyl)-3,7-dioxo-12,15,18-trioxa-2,8-diazahenicosan-21-yl)carbamate (**e**)[44]

To a solution of 5-((4-(6-methyl-1,2,4,5-tetrazin-3-yl)benzyl)amino)-5-oxopentanoic acid **c** (130 mg, 0.412 mmol), HATU (156 mg, 0.412 mmol), NEt_3 (57 µL, 0.412 mmol) in DCM (3 mL), and *tert*-butyl (3-(2-(2-(3-aminopropoxy)ethoxy)ethoxy)-propyl)carbamate **d** (131 mg, 0.45 mmol) in DCM (3 mL) was added. The resulting solution was stirred at 21 ºC for 16 h. The solvent was removed in vacuo, and the mixture re-dissolved in 1 M HCl solution (20 mL) and washed with DCM (3 × 20 mL) to remove unreacted 5-((4-(6-methyl-1,2,4,5-tetrazin-3-yl)benzyl)amino)-5-oxopentanoic acid. The aqueous phase was then basified with saturated aqueous K_2CO_3 solution, extracted with DCM (3 × 20 mL). The combined organic phases were washed with brine (20 mL), dried ($MgSO_4$), filtered and the solvent removed *in vacuo*. The crude residue was purified by flash column chromatography (0–10% MeOH in EtOAc) to afford *tert*-butyl (1-(4-(6-methyl-1,2,4,5-tetrazin-3-yl)phenyl)-3,7-dioxo-12,15,18-trioxa-2,8-diazahenicosan-21-yl)carbamate **e** (204 mg, 79%) as a purple oil. ^{1}H NMR (600 MHz, $CDCl_3$) δ 8.49 (d, J = 8.4 Hz, 2H), 7.53 (d, J = 8.5 Hz, 2H), 4.48-4.45 (m, 2H), 3.63-3.48 (m, 12H), 3.26-3.24 (m, 2H), 3.11-3.09 (m, 2H), 3.02 (s, 3H), 2.31 (t, J = 7.5 Hz, 2H), 2.23 (t, J = 6.9 Hz, 2H), 1.96 (p, J = 6 Hz, 2H), 1.77-1.68 (m, 4H), 1.41 (s, 9H); ^{13}C NMR (150 MHz, $CDCl_3$) δ 175.3 (C), 175.1 (C), 168.6 (C), 165.1(C), 158.4 (C), 145.0 (C), 132.2 (C), 129.2 (CH), 128.9 (CH), 79.7 (CH2), 71.4 (CH2), 71.4 (CH2), 71.1 (CH2 × 2), 69.8 (CH2), 69.8 (CH2), 43.7 (CH2), 38.6 (CH2), 37.7(CH2), 36.2 (CH2), 36.1 (CH2), 30.8 (CH2), 30.3 (CH2), 28.7 (CH2), 23.1 (CH3), 23.1 (C) 20.9 (CH3). IR (thin film) 3281, 3270, 3024, 2973, 2928, 2867,1692, 1628, 1523 cm^{-1}.

N[1]-(17-(4,5-dibromo-2-methyl-3,6-dioxo-3,6-dihydropyridazin-1(*2H*)-yl)-15-oxo-4,7,10-trioxa-14-azaheptadecyl)-*N*[5]-(4-(6-methyl-1,2,4,5-tetrazin-3-yl)benzyl)glutaramide (**2**)[45]

tert-butyl (1-(4-(6-methyl-1,2,4,5-tetrazin-3-yl)phenyl)-3,7-dioxo-12,15,18-trioxa-2,8-diazahenicosan-21-yl)carbamate **e** (136 mg, 0.231 mmol) was dissolved in a mixture of TFA and DCM (1:1, 4 mL) and the solution was stirred at room temperature for 90 min. The solvent was then removed in vacuo and the mixture re-dissolved in $NaHCO_3$ until no more gas is released. The reaction mixture is extracted with DCM (5 × 20 mL). The combined organic phases are dried ($MgSO_4$), filtered and the solvent is removed *in vacuo*. To the reaction mixture re-dissolved in DCM (3 mL) were added HATU (54 mg, 0.142 mmol) and NEt_3 (20 µL, 0.142 mmol) and was left to stir for 5 min at room temperature. After this time, the reaction mixture was added to a flame-dry flask with 5-((4-(6-methyl-1,2,4,5-tetrazin-3-yl)benzyl)amino)-5-oxopentanoic acid **f** (50 mg, 0.140 mmol) and the reaction mixture was left to stir for 16 h at room temperature. After this time, the reaction was concentrated in vacuo and the crude residue dissolved in $CHCl_3$ (25 mL) and washed with water (2 × 15 mL) and sat. aq. K_2CO_3 (15 mL). The organic layer was then dried ($MgSO_4$), filtered and the solvent removed *in vacuo*. Purification of the crude residue by flash column chromatography (5% to 20% MeOH/EtOAc) afforded compound **(2)** (43 mg, 50.3 µmol, 36%) as a purple oil. ^{1}H NMR (600 MHz, $CDCl_3$) δ 8.53 (d, J = 8.4 Hz, 2H), 7.50 (d, J = 8.5 Hz, 2H), 7.04 (t, J= 5.2 Hz, 1H), 6.80 (t, J = 5.8 Hz, 1H), 6.46 (t, J = 5.3 Hz, 1H), 4.55 (d, J = 6.0 Hz, 2H), 4.39 (t, J = 6.9 Hz, 2H), 3.69 (s, 3H), 3.63–3.50 (m, 12H), 3.36–3.26 (m, 4H), 3.09 (s, 3H), 2.80 (d, J = 4.8 Hz, 1H), 2.56 (t, J = 6.9 Hz, 2H), 2.36 (t, J = 7.3 Hz, 2H), 2.26 (t, J = 6.9 Hz, 2H), 1.99 (app. quint., J = 7.1 Hz, 2H), 1.78–1.69 (m, 4H); ^{13}C NMR (150 MHz, $CDCl_3$) δ 173.0 (C), 172.8 (C), 169.2 (C), 167.4 (C), 164.0 (C), 153.1 (C), 152.9 (C), 143.6 (C), 136.4 (C), 135.4 (C), 131.0 (C), 128.6 (2×CH), 128.3 (2×CH), 70.6 (2×CH2), 70.1 (2×CH2), 70.0 (2×CH2), 44.6 (CH2), 43.3 (CH2), 38.2 (CH2), 38.0 (CH2), 35.6 (CH2), 35.4 (CH2), 35.2 (CH3), 34.1 (CH2), 29.1 (CH2), 28.9 (CH2), 22.2 (CH2), 21.3 (CH3); IR (thin film) 3310, 2923, 2851, 1734, 1631, 1543 cm^{-1}; LRMS (ESI). 858 (50, $[M^{81}Br^{81}Br+H]^+$), 856 (100, $[M^{79}Br^{81}Br+H]^+$), 854 (50, $[M^{79}Br^{79}Br+H]^+$); HRMS (ESI) calcd for $C_{33}H_{46}Br_2N_9O_8Na$ $[M^{79}Br^{81}Br+Na]^+$ 878.1630; observed 878.1639.

In vitro evaluation of the bispecific antibodies

Cell culture

Jurkat cells and HCC1954 cells were cultured in RPMI 1640 supplemented with 10% v/v FBS, 50 units/mL penicillin and 50 µg/mL streptomycin. Primary human T-cells were isolated from the buffy coat fractions of anonymized healthy blood donors after obtaining ethical approval from the Queen's University Belfast Research Ethics Committee/Northern Ireland Blood Transfusion Service. Purified T-cells were cultured in RPMI 1640 supplemented with 10% v/v FBS, 50 units/mL penicillin and 50 µg/mL streptomycin. All cells were maintained in 5% CO_2 at 37 °C in a humidified incubator.

Flow cytometry

A total of 2.5 x 10^5 HCC1954 cells or Jurkat cells were resuspended in 1 mL media containing 2.5 nM of the bispecific antibody or control and incubated for 1 h at 4 °C. Cells were then washed by centrifugation at 600 × g for 5 min at 4 °C and resuspended in 1 mL FACS buffer (5% v/v FBS in PBS) and centrifuged again. The cells were resuspended in 100 µL FACS buffer containing 1.25 ug/mL FITC anti-human IgG Fc antibody. Following incubation for 30 mins at 4 °C in dark, cells were washed twice in FACS buffer by centrifugation at 600 × g for 5 mins at 4 °C, resuspended in PBS buffer and fluorescence was assessed on a BD Accuri C6 Plus flow cytometer.

T-cell purification

Healthy donor buffy coats were mixed with an equal volume of Hank's Balanced Salt Solution (HBSS) and 30 mL aliquots of diluted blood were gently layered over 15 mL Lymphoprep™ density gradient medium in 50 mL conical tubes, followed by centrifugation at 800 × g for 30 min at room temperature without brake. The peripheral blood mononuclear cell (PBMC) fraction was transferred to a fresh tube, resuspended in 50 mL HBSS and centrifuged at 300 × g for 10 min at room temperature. To facilitate platelet removal, this HBSS resuspension-centrifugation step was repeated twice at a lower speed of 200 × g. T cells were then purified from the PBMC fraction by negative selection using the EasySep™ Human T Cell Isolation Kit (STEMCELL Technologies) in accordance with the manufacturer's instructions.

T-cell/HCC1954 cell coculture

HCC1954 cells were seeded at 5 x 10^3 per well in 96-well plates and left to adhere overnight. Purified T-cells were then added to appropriate wells at an effector:target (E:T) ratio of 10:1 followed by 5 nM treatment. After 48 h, culture supernatants were collected and centrifuged to remove cells. Cell-free supernatants were stored at -80 °C in preparation for downstream ELISA analysis. Microplate wells were then used to assess the viability of remaining adherent HCC1954 cells by CellTiter-Glo® assay in accordance with the manufacturer's instructions.

ELISA

Cell-free culture supernatants were assayed using the human IFN-γ DuoSet ELISA (R&D Systems) in accordance with the manufacturer's instructions.

Dose-response study

HCC1954 cells were seeded at 5 x 10^3 per well in 96-well plates and left to adhere overnight. Purified T-cells were then added to appropriate wells at an effector:target (E:T) ratio of 10:1 followed by the addition of varying concentrations (0.01289 pM to 5 nM) of the treatment. After 48 h, culture supernatants were removed and the microplate wells were used to assess the viability of remaining adherent HCC1954 cells by CellTiter-Glo® assay in accordance with the manufacturer's instructions.

Data analysis

FlowJo software (version 10.8.1) was used to construct histograms in Figure 3A. GraphPad Prism software (version 9.3.1) was used to graph data.

NMR spectra

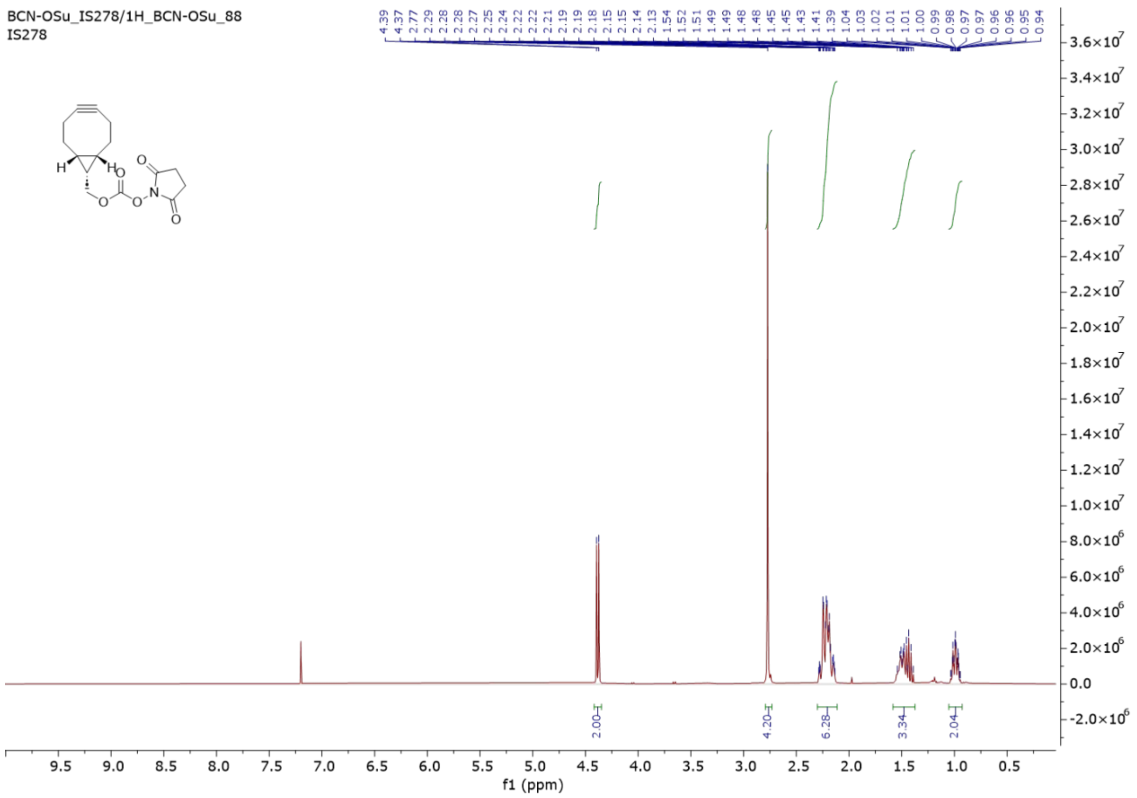

Fig. S14: ^{1}H NMR spectrum of BCN-OSu.

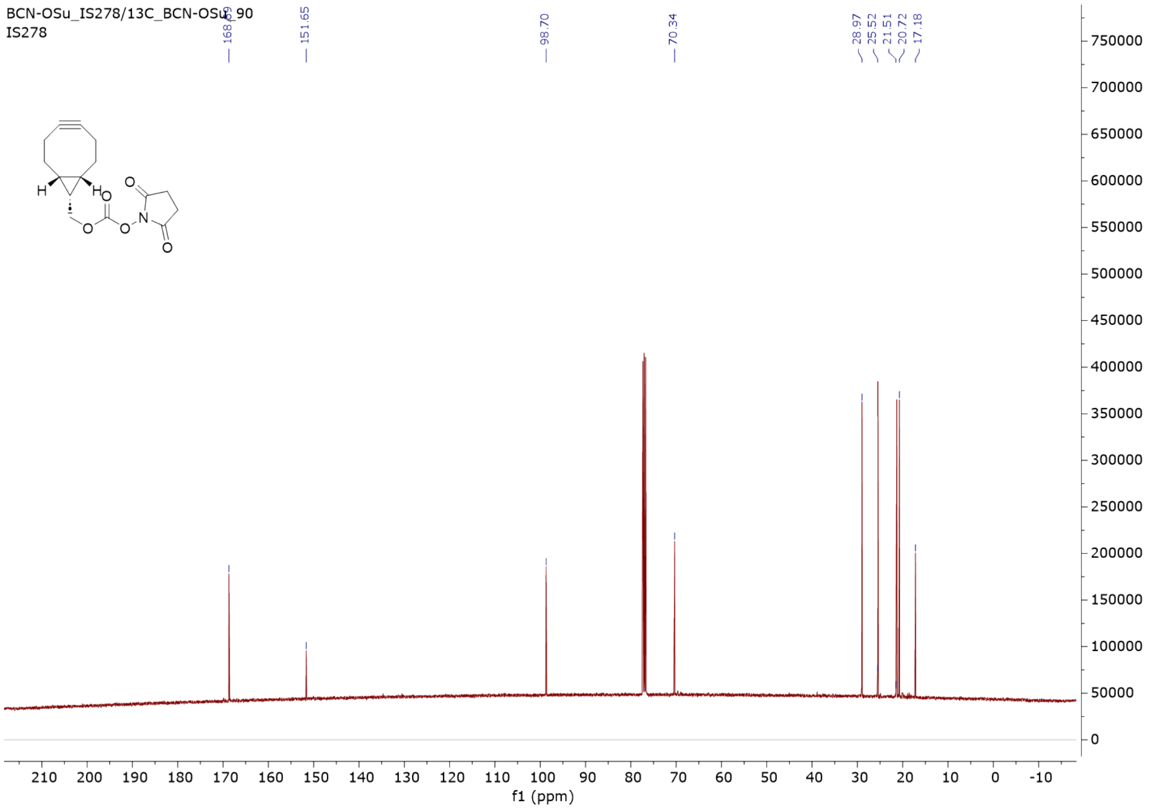

Fig. S15: ^{13}C NMR spectrum of BCN-OSu.

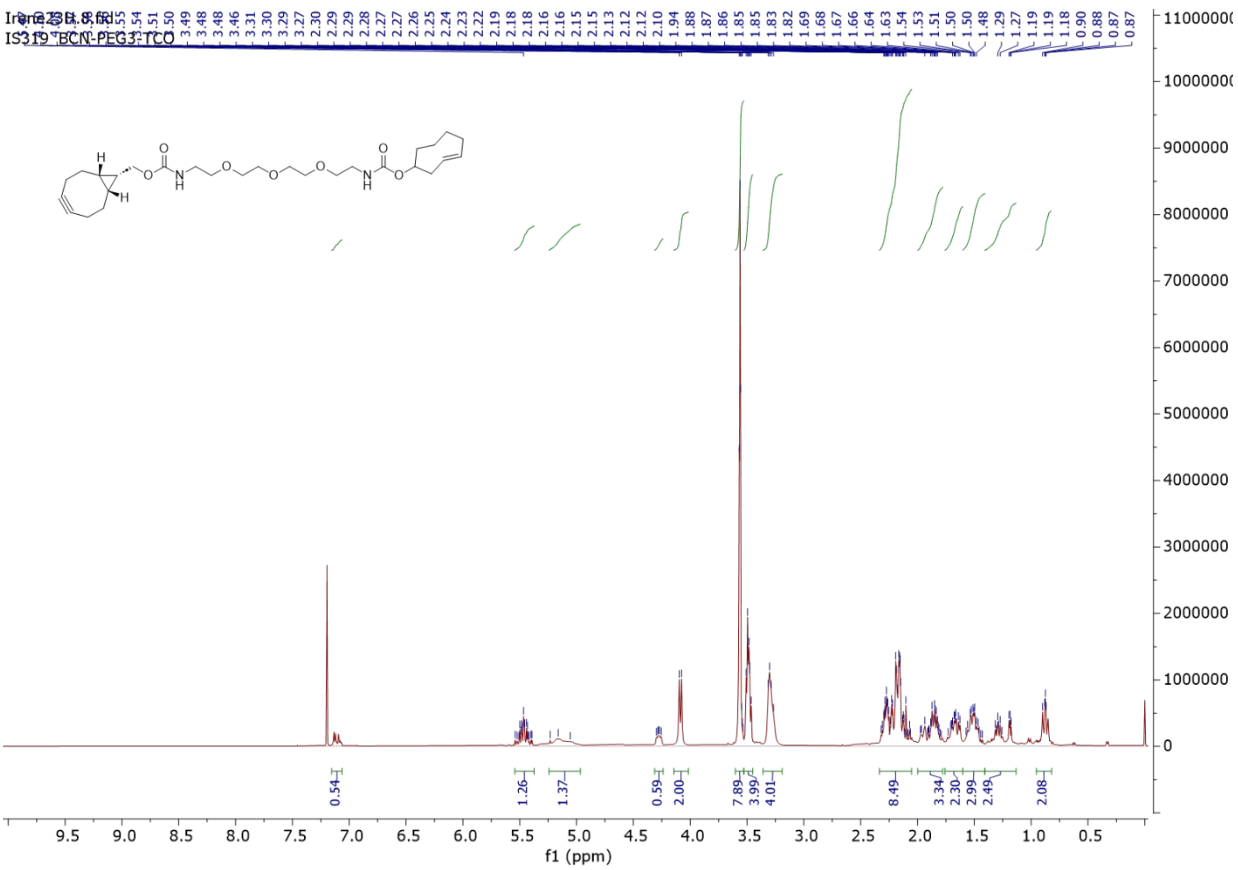

Fig. S16: ^{1}H NMR spectrum of BCN-PEG$_3$-TCO (**1**).

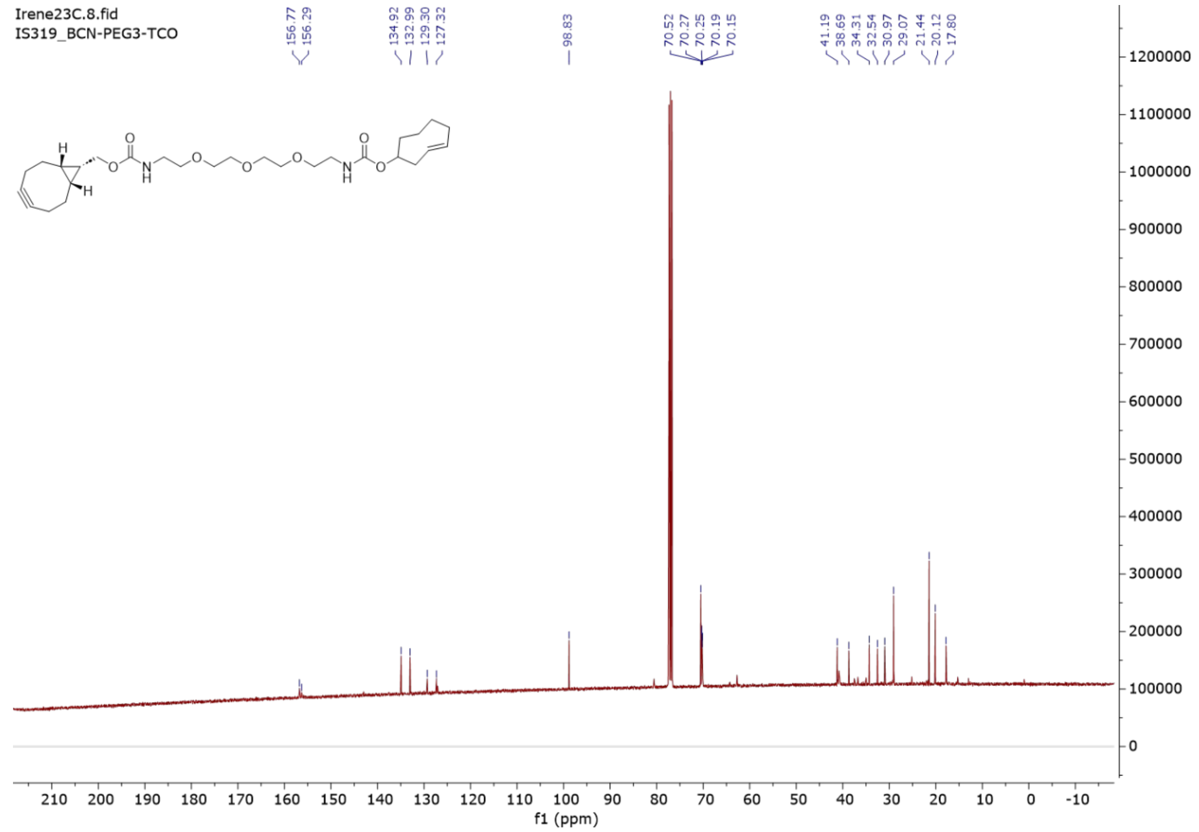

Fig. S17: ^{13}C NMR of BCN-PEG$_3$-TCO (**1**).

LC-MS data

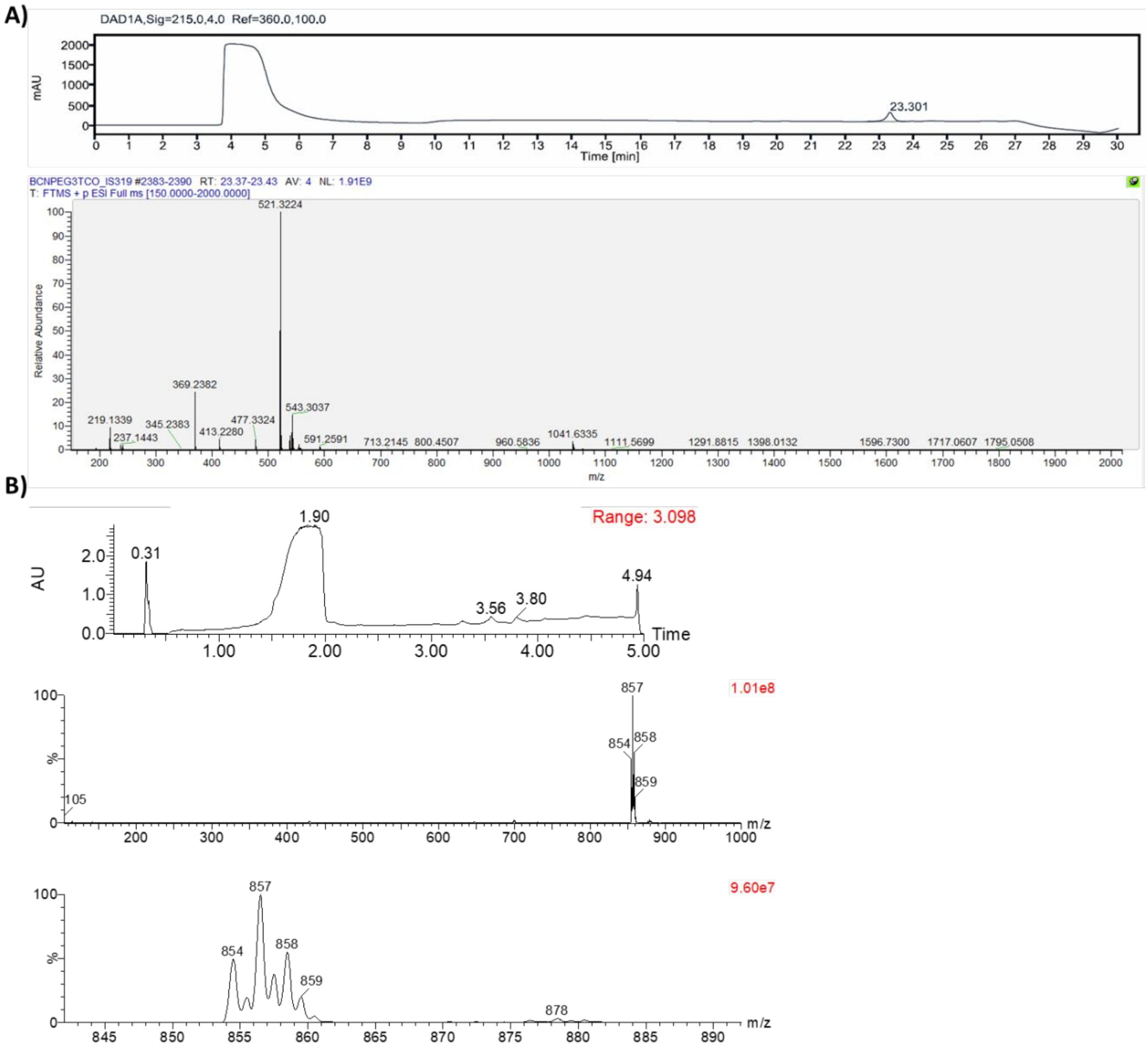

Fig. S18: A) HPLC-UV trace and MS trace of BCN-PEG3-TCO (**1**), and B) HPLC-UV trace and MS trace of Br_2PD-Tz (**2**).

SEC-nMS of the antibody conjugates

Native SEC-MS measurements were performed on an Acquity UPLC-H-class system (Waters, Wilmslow, UK) comprising a quaternary solvent manager, a sample manager at 10°C, a column oven at room temperature and a TUV detector operating at 214 nm and 280 nm, coupled to a Q-TOF Synapt G2 HDMS mass spectrometer (Waters, Manchester, UK). Ten to twenty µg of mAb samples were injected through a MaxPeak Premier Protein SEC 250Å, 1.7 µm, 4.6 x 150 mm column (Waters, Manchester, UK) using an isocratic gradient of 150 mM $AcNOH_4$ (pH 6.9) at a flowrate of 250 µL/min over 6 min. The Synapt G2 was calibrated and tuned using a 2 g/L solution of cesium iodide in 2-propanol/water (50/50 v/v), and then was operated in sensitivity mode using a + 3.0 kV capillary voltage. The backing pressure (Pi) and the sample cone voltage (Vc) were set at, 6 mbar and 180 V, respectively. The acquisitions were performed on an m/z range of 1000-10,000 with a 1.5 s scan time. MS data processing was performed using Mass Lynx V4.1 (Waters, Manchester, UK). To relatively quantify the baseline unresolved species, we performed a Gaussian fitting in Igor Pro v6.05. Experimental data were collected from the UV measurements of HER2xHER2 and HER2xCD3 bispecific constructs and were imported into the software. The experimental data showed two overlapping peaks for each sample in the (3.7-4.4 min) region. Based on this data, a Gaussian function, representing the fitting model of each peak in the overlapping region, was defined with two parameters: retention time and full width at half maximum (FWHM). The Gaussian fit was adjusted to provide a perfect match between the fitting model and the experimental data (R^2=0.997). After assessing the quality of the fit Gaussian models, the relative quantification was performed based on the peak areas (see Figure 1 in the manuscript).

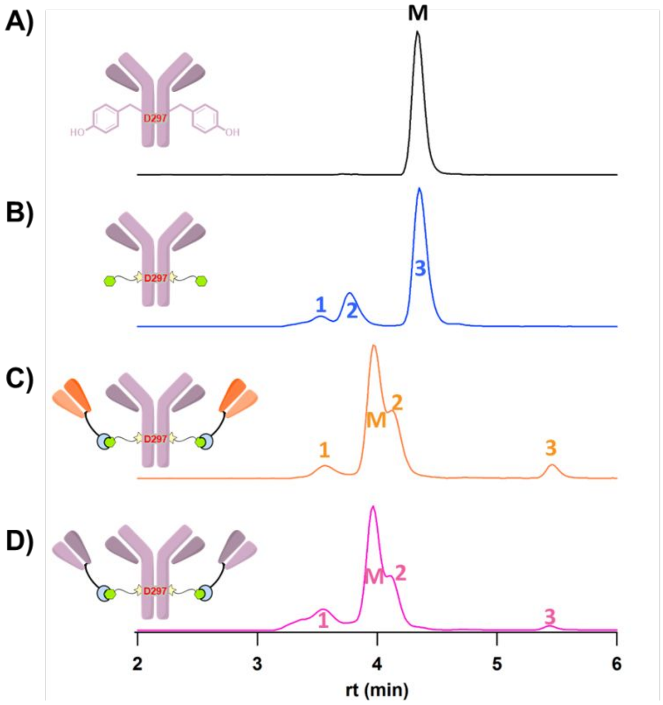

Peak	Species	t_R (min)	Experimental mass (Da)
Main peak	Deglycosylated Trz	4.33	145 175 ± 1 Da
Peak 1	(Trz-TCO)$_3$	3.52	438 902 ± 30 Da
Peak 2	(Trz-TCO)$_2$	3.77	292 495 ± 12 Da
Main peak	(Trz-TCO)$_1$	4.35	146 248 ± 1 Da
Peak 1	N.A	3.62	N.A
Main peak	HER2xCD3 2:2 (**5a**)	3.95	242 491 ± 4 Da
Peak 2	HER2xCD3 2:1	4.12	194 386 ± 4 Da
Peak 3	Fab$_{CD3}$	5.43	48 142 ± 1 Da
Peak 1	N.A	3.53	283 939 ± 10 Da
Main peak	HER2xHER2 2:2 (**5b**)	3.97	242 889 ± 4 Da
Peak 2	HER2xHER2 2:1	4.12	194 581 ± 3 Da
Peak 3	Fab$_{HER2}$	5.43	48 333 ± 1 Da

Fig. S19: SEC-UV chromatograms of various synthetic components: A) deglycosylated trastuzumab, B) trastuzumab-TCO (**4**), C) HER2xCD3 bsAc (**5a**), D) tetravalent HER2 construct (**5b**). N.A. = not assigned, which indicates that this compound does not appear to be related to a mAb (fragment).

References

(39) Marty, M. T.; Baldwin, A. J.; Marklund, E. G.; Hochberg, G. K. A.; Benesch, J. L. P.; Robinson, C. V. Bayesian Deconvolution of Mass and Ion Mobility Spectra: From Binary Interactions to Polydisperse Ensembles. *Anal. Chem.* **2015**, *87*, 4370–4376. https://doi.org/10.1021/acs.analchem.5b00140.

(40) Lang, K.; Davis, L.; Wallace, S.; Mahesh, M.; Cox, D. J.; Blackman, M. L.; Fox, J. M.; Chin, J. W. Genetic Encoding of Bicyclononynes and Trans-Cyclooctenes for Site-Specific Protein Labeling in Vitro and in Live Mammalian Cells via Rapid Fluorogenic Diels-Alder Reactions. *J. Am. Chem. Soc.* **2012**, *134*, 10317–10320. https://doi.org/10.1021/ja302832g.

(41) Yang, J.; Karver, M. R.; Li, W.; Sahu, S.; Devaraj, N. K. Metal-Catalyzed One-Pot Synthesis of Tetrazines Directly from Aliphatic Nitriles and Hydrazine. *Angew. Chem. Int. Ed.* **2012**, *51*, 5222–5225. https://doi.org/10.1002/anie.201201117.

(42) Hernández-Gil, J.; Braga, M.; Harriss, B. I.; Carroll, L. S.; Leow, C. H.; Tang, M. X.; Aboagye, E. O.; Long, N. J. Development of 68Ga-Labelled Ultrasound Microbubbles for Whole-Body PET Imaging. *Chem. Sci.* **2019**, *10*, 5603–5615. https://doi.org/10.1039/c9sc00684b.

(43) Lee, M. T. W.; Maruani, A.; Richards, D. A.; Baker, J. R.; Caddick, S.; Chudasama, V. Enabling the Controlled Assembly of Antibody Conjugates with a Loading of Two Modules without Antibody Engineering. *Chem. Sci.* **2017**, *8*, 2056–2060. https://doi.org/10.1039/c6sc03655d.

(44) Nogueira, C. J. F. Forming Next-Generation Antibody-Nanoparticle Conjugates through the Oriented Installation of Antibody Fragments. Ph.D Dissertation, University College London, United Kingdom, 2019. https://discovery.ucl.ac.uk/id/eprint/10086139/ (accessed 2023-07-12)

(45) Maruani, A.; Szijj, P. A.; Bahou, C.; Nogueira, J. C. F.; Caddick, S.; Baker, J. R.; Chudasama, V. A Plug-and-Play Approach for the de Novo Generation of Dually Functionalized Bispecifics. *Bioconjug. Chem.* **2020**, *31*, 520–529. https://doi.org/10.1021/acs.bioconjchem.0c00002.
